# Investigation of Multi-Input Multi-Output Robust Control Methods to Handle Parametric Uncertainties in Autopilot Design

**DOI:** 10.1371/journal.pone.0165017

**Published:** 2016-10-26

**Authors:** Coşku Kasnakoğlu

**Affiliations:** Department of Electrical and Electronics Engineering, TOBB University of Economics and Technology, Ankara, Turkey; Chongqing University, CHINA

## Abstract

Some level of uncertainty is unavoidable in acquiring the mass, geometry parameters and stability derivatives of an aerial vehicle. In certain instances tiny perturbations of these could potentially cause considerable variations in flight characteristics. This research considers the impact of varying these parameters altogether. This is a generalization of examining the effects of particular parameters on selected modes present in existing literature. Conventional autopilot designs commonly assume that each flight channel is independent and develop single-input single-output (SISO) controllers for every one, that are utilized in parallel for actual flight. It is demonstrated that an attitude controller built like this can function flawlessly on separate nominal cases, but can become unstable with a perturbation no more than 2%. Two robust multi-input multi-output (MIMO) design strategies, specifically loop-shaping and *μ*-synthesis are outlined as potential substitutes and are observed to handle large parametric changes of 30% while preserving decent performance. Duplicating the loop-shaping procedure for the outer loop, a complete flight control system is formed. It is confirmed through software-in-the-loop (SIL) verifications utilizing blade element theory (BET) that the autopilot is capable of navigation and landing exposed to high parametric variations and powerful winds.

## Introduction

Quite a few applications require automatic flight control techniques that help or even replace the human pilot [1, 2]. Autopilot technologies have advanced significantly over time to present-day autopilots that achieve path following and landing rather proficiently [3–5]. Autopilot technologies are no longer reserved to military fighters and large-airlines; coupled with the increased availability of unmanned aerial vehicles (UAVs) to the common researcher, they have found application in many smaller-scale civilian projects [6–8]. Procedures utilized for autopilot design comprise of dynamic inversion [9], nonlinear optimal predictive control [10], reconfigurable flight control laws [11], robust nonlinear control [12], Lyapunov vector fields [13], command filtered backstepping [14], sliding mode control [15], multiple model adaptive control [16], invariant manifolds [17], fault tolerant *H*_∞_ control [18] and geometric control [19].

Conventional autopilot models usually approximate every flight channel as independent and create single-input single-output (SISO) controllers [2]. To regulate the banking angle for instance, a SISO model from aileron deflection to roll angle is formulated, on which a SISO control technique (e.g. PID) is employed [4]. The operation is continued for every aspect being controlled and all the SISO designs are finally used concurrently in the ultimate design. This method disregards the couplings amongst the channels. However, these side effects are usually not powerful in most fixed-winged airplanes so it continues to remain a dominant approach. Multi-input multi-output (MIMO) methods are usually preferred for missiles [20–23], helicopters [24–26] and multirotor vehicles [27–30] where dynamical couplings are dominant.

Regrettably, numerous research work hint that there exist threats linked to the standard SISO design method for even fixed-winged aerial vehicles. High crosswinds, aerobatics, structural damage and actuator failures could lead to the aircraft getting forced away from its normal behaviour [31, 32]. These kinds of scenarios break the regular weak-coupling suppositions. It is furthermore common in conventional SISO designs to presume that the airplane variables like geometry, mass, inertias and stability derivatives are known completely. In actuality it is hard to come by accurate estimates to these variables. In reports assessing popular methods such as Advanced Aircraft Analysis (AAA), Athena Vortex Lattice (AVL), wind tunnel based modelling and real flight dependent modelling, it has been discovered that one method could possibly produce parameter values quite different from an alternative approach [33–36]. These parameters get inserted inside the system model during linearization of the nonlinear dynamics about a particular operating condition. A controller constructed on this sort of a model is then utilized for the whole flight envelope. This often works nicely for the nominal case yet performance degrades and destabilization can take place for perturbed conditions [37].

The two primary cases for which departure occurs from the nominal circumstances are leaving the vicinity of the operating point and the existence of uncertainties in the aircraft parameters. Although many results are documented with regards to the former [38, 39], it is hard to find a single study on the latter for the complete parameter set on the whole aircraft (i.e. not just particular parameters on particular modes [40]). In this paper a practical oriented study is given to investigate this issue. Via an example on a general aviation airplane, it is proven that the common technique of designing SISO controllers for each channel may have bad performance and even lose stability under modest parametric uncertainties. We then look at two robust MIMO design methods, namely loop-shaping and *μ*-synthesis, which can endure large parametric variations and at the same time maintain decent performance. Nonlinear simulations along with software-in-the-loop (SIL) verifications utilizing blade element theory (BET) [41], [42] are executed. BET is commonly perceived as an extremely reliable way of carrying out aerospace simulations in which all surfaces of the aircraft are represented as “blade elements” for force and moment calculations.

The scientific contribution of this work can be summarized under three items:

1. *Analysis of the critical effects of varying all aircraft parameters simultaneously*: In this work, all the parameters related to mass, geometry and the dynamical behavior of the aircraft are varied simultaneously to first uncover the possibility of fatal results with variations as little as 2% under traditional control approaches. These traditional approaches are presently embedded in many of the existing autopilot systems used in civilian and military aircraft.
2. *Systematic construction of autopilots to remedy the risks associated with parameter variations*: It is demonstrated that MIMO robust control approaches can endure large parametric variations and at the same time maintain good performance for aircrafts. Step by step construction using two such methods, namely loop-shaping and *μ*-synthesis, is outlined. It is seen that as much as 30% changes on the full parameter set is acceptable without significant sacrifice in stability and performance.
3. *Separation of design-models and verification-models*: A clear separation between the aircraft model used in design and that used for verification is made for this study. The autopilot designs utilize a mathematical model based on the theory of aircraft dynamics. This model consists of nonlinear differential equations for which the forces and moments are computed using coefficients called stability derivatives. Once the controllers are established, the final closed-loop verifications are done with SIL tests which are based on different and much more accurate models of the aircraft. These models use BET in which all surfaces of the aircraft are subdivided into small regions called blade elements for force and moment calculations. BET flight simulations are widely regarded to produce the most realistic results. Moreover, they are not based on ordinary differential equation models of the aircraft such as the one we use here for designing the controllers. Avoiding the same type of model in the testing phase is advantageous to increase the validity of the proposed methods.

The remainder of the paper is structured as follows: Section Methodology sets out the structure for the study together with background information on necessary tools. Section Results performs the analyses on a general aviation aircraft and presents the outcomes. Section Conclusions wraps up the article with conclusions and future research ideas.

## Methodology

### Mathematical Model

The first step is the derivation of the mathematical model on which numerical analysis and controller design will be carried out. The model of the aircraft dynamics is acquired from rigid body force and moment equations:

$$\begin{array}{cc} F & =m\left(\frac{\partial V}{\partial t}+\Omega \times V\right) \end{array}$$ (1)

$$\begin{array}{cc} M & =\frac{\partial (I\cdot \Omega )}{\partial t}+\Omega \times \left(I\cdot \Omega \right) \end{array}$$ (2)

where *V* = [*u* *v* *w*]^*T*^ is the velocity vector at the center of gravity, Ω = [*p* *q* *r*]^*T*^ is the angular velocity vector about the center of gravity, *F* = [*F*_*x*_ *F*_*y*_ *F*_*z*_]^*T*^ is the total external force vector, and *M* = [*L* *M* *N*]^*T*^ is the total external moment vector. *I* is the inertia tensor of the rigid body defined as

I=-Ixx-Jxy-Jxz-Jyx-Iyy-Jyz-Jzx-Jzy-Izz.

The coefficients of the matrix *I* are the moments and products of inertia of the rigid body and they are constant for a frame of reference fixed to the aircraft. A rearrangement of Eqs (1) and (2) yields

$$\begin{array}{cc} \frac{\partial V}{\partial t} & =\frac{F}{m}-\Omega \times V \end{array}$$ (3)

$$\begin{array}{cc} \frac{\partial (I\cdot \Omega )}{\partial t} & =M-\Omega \times (I\cdot \Omega ). \end{array}$$ (4)

After some manipulations the following non-linear state space system is obtained

$$\begin{array}{c} x=f(x,F(t),M(t)) \end{array}$$ (5)

with

$$\begin{array}{c} F=g_{1}\left(x\left(t\right),u\left(t\right),v\left(t\right),t\right) \end{array}$$ (6)

$$\begin{array}{c} M=g_{2}\left(x\left(t\right),u\left(t\right),v\left(t\right),t\right). \end{array}$$ (7)

These equations can be written compactly as

$$\begin{array}{c} x=f(x(t),u(t),v(t),t) \end{array}$$ (8)

with state vector *x*, input vector *u*, disturbance vector *v*, and time *t*. The state vector *x* normally consists of three linear and three angular velocities from *V* and Ω. For practical applications however, it is usually much easier to use airspeed, angle of attack and sideslip angle rather than the linear velocity components. This produces the state vector:

$$\begin{array}{c} x={\left[V\;\alpha \;\beta \;p\;q\;r\;\psi \;\theta \;\phi \;x_{e}\;y_{e}\;z_{e}\right]}^{T} \end{array}$$ (9)

in terms of which the state space equations can be derived as:

$$\begin{array}{c} \dot{V}=\frac{1}{m}\left(F_{x}\cos\alpha \cos\beta +F_{y}\sin\beta +F_{z}\sin\alpha \sin\beta \right) \end{array}$$

$$\begin{array}{l} \dot{\alpha }=\frac{1}{V\text{cos}\beta }\left[\frac{1}{m}(-F_{x}\text{sin}\alpha +F_{z}\text{cos}\alpha )\right]+q\\ \;-(p\text{cos}\alpha +r\text{sin}\alpha )\text{tan}\beta \end{array}$$

$$\begin{array}{l} \dot{\beta }=\frac{1}{V}\left[\frac{1}{m}\left(-F_{x}\text{cos}\alpha \text{sin}\beta +F_{y}\text{cos}\beta -F_{z}\text{sin}\alpha \text{sin}\beta \right)\right]\\ \;+p\text{sin}\alpha -r\text{cos}\alpha \end{array}$$

$$\begin{array}{l} \dot{p}=P_{pp}p^{2}+P_{pq}pq+P_{pr}pr+P_{qq}q^{2}+P_{qr}qr\\ \;+P_{rr}r^{2}+P_{l}L+P_{m}M+P_{n}N \end{array}$$

$$\begin{array}{l} \dot{q}=Q_{pp}p^{2}+Q_{pq}pq+Q_{pr}pr+Q_{qq}q^{2}+Q_{qr}qr\\ \;+Q_{rr}r^{2}+Q_{l}L+Q_{m}M+Q_{n}N \end{array}$$

$$\begin{array}{l} \dot{r}=R_{pp}p^{2}+R_{pq}pq+R_{pr}pr+R_{qq}q^{2}+R_{qr}qr\\ \;+R_{rr}r^{2}+R_{l}L+R_{m}M+R_{n}N \end{array}$$

$$\dot{\psi }=q\text{sin}\phi +r\text{cos}\phi \text{cos}\theta$$

$$\begin{array}{c} \dot{\theta }=q\cos\phi -r\sin\phi \end{array}$$

$$\begin{array}{c} \dot{\phi }=p+(q\sin\phi +r\cos\phi )\tan\theta \end{array}$$

$$\begin{array}{l} {\dot{x}}_{e}=\left[u\text{cos}\theta +\left(v\text{sin}\phi +w\text{cos}\phi \right)\text{sin}\theta \right]\text{cos}\psi \\ \;-\left(v\text{cos}\phi -w\text{sin}\phi \right)\text{sin}\psi \end{array}$$

$$\begin{array}{l} {\dot{y}}_{e}=\left[u\text{cos}\theta +\left(v\text{sin}\phi +w\text{cos}\phi \right)\text{sin}\theta \right]\text{sin}\psi \\ \;+\left(v\text{cos}\phi -w\text{sin}\phi \right)\text{cos}\psi \end{array}$$

where

$${\dot{z}}_{e}=u\text{sin}\theta -\left(v\text{sin}\phi +w\text{cos}\phi \right)\text{cos}\theta$$

$$\dot{u}=\frac{F_{x}}{m}-qw+rv$$

$$\dot{v}=\frac{F_{y}}{m}+pw-ru$$

$$\dot{w}=\frac{F_{z}}{m}-pv+qu$$

and *P*_*pp*_, *P*_*pq*_, *P*_*pr*_, *P*_*qq*_, *P*_*qr*_, *P*_*rr*_, *P*_*l*_, *P*_*m*_, *P*_*n*_, *Q*_*pp*_, *Q*_*pq*_, *Q*_*pr*_, *Q*_*qq*_, *Q*_*qr*_, *Q*_*rr*_, *Q*_*l*_, *Q*_*m*_, *Q*_*n*_, *R*_*pp*_, *R*_*pq*_, *R*_*pr*_, *R*_*qq*_, *R*_*qr*_, *R*_*rr*_, *R*_*l*_, *R*_*m*_, *R*_*n*_ are values determined by the inertia values. The equations given listed here are in the East, North, Up (ENU) reference frame. The sign of ${\dot{z}}_{e}$ can be reversed if North, East, Down (NED) is desired. For solving the abovementioned differential equations one needs to get the force and moment values *F* = [*F*_*x*_ *F*_*y*_ *F*_*z*_]^*T*^ and *M* = [*L* *M* *N*]^*T*^. These depend on a variety of mass and geometry parameters, the thrust mechanism, along with the control commands. These forces and moments are handily depicted with respect to stability derivatives, which capture the impact of various important variables on a given force or moment value. For example, the longitudinal aerodynamical force can be written as

$$\begin{array}{l} F_{x}=C_{X_{0}}+C_{X_{\alpha }}\alpha +C_{X_{\alpha^{2}}}\alpha^{2}+C_{X_{\alpha^{3}}}\alpha^{3}\\ \;+C_{X_{q}}\frac{q\bar{c}}{V}+C_{X_{\delta_{r}}}\delta_{r}+C_{X_{\delta_{f}}}\delta_{f}+C_{X_{\alpha \delta_{f}}}\alpha \delta_{f} \end{array}$$ (10)

where *C*_*X*_0__, *C*_*X*_*α*__, $C_{X_{\alpha^{2}}}$, $C_{X_{\alpha^{3}}}$, *C*_*X*_*q*__, $C_{X_{\delta_{r}}}$, $C_{X_{\delta_{r}}}$, $C_{X_{\alpha \delta_{f}}}$ are the stability derivatives capturing the effect of the term which they multiply on *F*_*x*_. Expressions for *F*_*y*_, *F*_*z*_, *L*, *M*, *N* may be expressed in a similar fashion in terms of their matching stability derivatives [43].

For the implementation of the mathematical model we utilize the Flight Dynamics & Control (FDC) Toolbox for MATLAB [43]. The simulation results produced by this package were tested on real aircraft with successful results. While a different aircraft was used in the FDC Toolbox, actual values for mass, geometry and stability derivatives for Cessna 172 are available in various reliable sources including reports form Cessna Aircraft Company itself [44], making it possible to obtain a valid and reliable Cessna 172 model.

It is difficult to perform a direct validation of the aircraft model based on real flight data since we do not currently have access to a real Cessna 172 and a flight recorder. Nor were we unable to locate any work in literature where such data is openly available. The closest is the study of Neuhart et al. [45] where actual flight data from a Cessna 172 was collected using a custom-built data acquisition system. While the comprehensive data set is not provided numerically, a scenario involving a stall-test is presented through some plots. In this scenario the aircraft was trimmed in a wings-level attitude for 30° flap with a fixed throttle setting. The pilot increased elevator input until stall was achieved. This test matches JAA test 2c8 and FAA test 2c9 [46, 47]. Although numerical data for this test is not available, looking at the figures one can eyeball that the aircraft stalls when the elevator input is around 15°.

**Fig 1:**
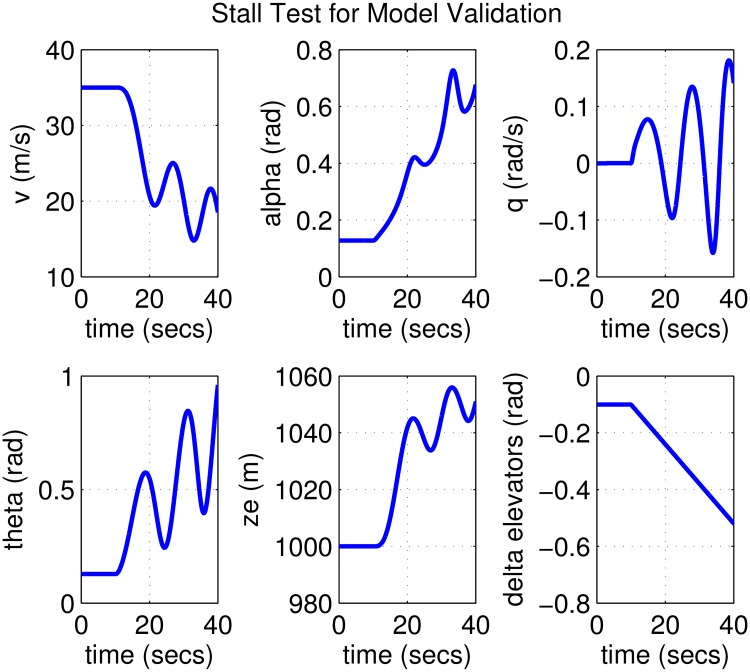
Stall test with 30° flap for model validation with stall occurring around *t* = 20 s.

For validation purposes we make an attempt to replicate the scenario above with our mathematical model as follows: We trim the Cessna 172 model with 30° flap for straight and level flight, after which the elevator input is increased gradually until stall happens. The results can be seen in Fig 1 where it is observed that the aircraft stalls around roughly *t* = 20 s, when the elevator is at 0.25 rad = 14.324°. This is quite close to the 15° observed in actual flight [45]. This serves as an additional proof of our model’s acceptable accuracy, as well as the feasibility and effectiveness of the analysis and control design to be carried out on it.

### Trimming and Linearization

Trimming describes the procedure for identifying an operating point for a provided flight condition. In our design, the aim is to solve the following equations

$$\begin{array}{c} V=V_{0},\;z_{e}=z_{e,0}, \end{array}$$ (11)

$$\begin{array}{cc} \frac{\partial \alpha }{\partial t} & =\frac{\partial \beta }{\partial t}=\frac{\partial p}{\partial t}=\frac{\partial q}{\partial t}=\frac{\partial r}{\partial t}=0, \end{array}$$ (12)

$$\begin{array}{cc} \frac{\partial \psi }{\partial t} & =\frac{\partial \theta }{\partial t}=\frac{\partial \psi }{\partial t}=\frac{\partial z_{e}}{\partial t}=0 \end{array}$$ (13)

for

$$\begin{array}{c} x={\left[V\;\alpha \;\beta \;p\;q\;r\;\psi \;\theta \;\phi \;x_{e}\;y_{e}\;z_{e}\right]}^{T}, \end{array}$$ (14)

$$\begin{array}{c} u={\left[F_{x}\;\delta_{e}\;\delta_{a}\;\delta_{r}\right]}^{T} \end{array}$$ (15)

where *V*_0_ is the chosen airspeed, *z*_*e*, 0_ is the desired attitude and the time derivatives are calculated from the nonlinear dynamical equations provided in Section Mathematical Model. The trimming procedure may be presented as the subsequent general optimization problem as

$$\begin{array}{c} \min_{x,u}f\left(x,u\right) \end{array}$$ (16)

subject to

$$\begin{array}{c} g_{1}\left(x,u\right)=0,\;g_{2}\left(x,u\right)=0 \end{array}$$ (17)

where

$$\begin{array}{cc} & f\left(x,u\right)={\left(\frac{\partial \alpha }{\partial t}\right)}^{2}+{\left(\frac{\partial \beta }{\partial t}\right)}^{2}+{\left(\frac{\partial p}{\partial t}\right)}^{2}+{\left(\frac{\partial q}{\partial t}\right)}^{2}+\\ & {\left(\frac{\partial r}{\partial t}\right)}^{2}+{\left(\frac{\partial \psi }{\partial t}\right)}^{2}+{\left(\frac{\partial \theta }{\partial t}\right)}^{2}+{\left(\frac{\partial \phi }{\partial t}\right)}^{2}+{\left(\frac{\partial z_{e}}{\partial t}\right)}^{2} \end{array}$$

and

$$\begin{array}{c} g_{1}\left(x,u\right)=V-V_{0},\;g_{2}\left(x,u\right)=z_{e}-z_{e,0}. \end{array}$$

Ideally *f*(*x*, *u*) = 0 yet because of numerical implementation and round-off errors *f*(*x*, *u*) < 10^−3^ is appropriate in reality. When the problem is formed as an optimization problem as above, it can be solved by means of effective numerical methods like Sequential Quadratic Programming (SQP) [48, 49]. The trim point solving the optimization problem in Eqs (16) and (17) is expressed as (*x*_0_, *u*_0_) where *x*_0_ is vector of the aircraft states at the operating condition and *u*_0_ is the vector of control inputs to be applied at the trim condition. The nonlinear aircraft model could then be linearized around the operating conditions (*x*_0_, *u*_0_), which produces a linear state-space system *G* of the form to be utilized in controller design

$$\begin{array}{c} G:\left\{\begin{array}{c} \dot{\tilde{x}}=A\tilde{x}+B\tilde{u}\\ y=C\tilde{x}+D\tilde{u} \end{array}\right. \end{array}$$ (18)

where $\tilde{x}=x-x_{0}$ $\tilde{u}=u-u_{0}$ and

$$\begin{array}{c} A=\frac{\partial f}{\partial x}\left(x_{0},u_{0}\right),\;B=\frac{\partial f}{\partial u}\left(x_{0},u_{0}\right), \end{array}$$ (19)

$$\begin{array}{c} C=\frac{\partial h}{\partial x}\left(x_{0},u_{0}\right),\;D=\frac{\partial h}{\partial u}\left(x_{0},u_{0}\right). \end{array}$$ (20)

The vectors fields *f*(*x*, *u*) and *h*(*x*, *u*) consist of respectively the equations for derivatives of the states and the outputs to be controlled. The linearized system may also be written in transfer function matrix form

$$\begin{array}{c} G\left(s\right)=C{\left(sI-A\right)}^{-1}B+D \end{array}$$ (21)

where *I* is the identity matrix.

### *H*_∞_ Loop-shaping

The first MIMO control strategy utilized in this study is *H*_∞_ loop-shaping. In loop-shaping control design, the desired specifications are commonly expressed as

$$\begin{array}{c} \bar{\sigma }\left(S\left(j\omega \right)\right)\leq |W_{1}^{-1}\left(j\omega \right)| \end{array}$$ (22)

$$\begin{array}{c} \bar{\sigma }\left(T\left(j\omega \right)\right)\leq |W_{3}^{-1}\left(j\omega \right)| \end{array}$$ (23)

where $\underset{\bar{}}{\sigma }$ and $\bar{\sigma }$ denote minimum and maximum singular values respectively. Here, *S*(*s*) is the sensitivity function defined as

$$\begin{array}{c} S\left(s\right)={\left(I+L\left(s\right)\right)}^{-1}, \end{array}$$ (24)

*T*(*s*) is the complementary sensitivity function defined as

$$\begin{array}{c} T\left(s\right)=L\left(s\right){\left(I+L\left(s\right)\right)}^{-1}, \end{array}$$ (25)

*L*(*s*) is the loop transfer function matrix

$$\begin{array}{c} L(s)=G(s)K(s), \end{array}$$ (26)

$|W_{1}^{-1}\left(j\omega \right)|$ is the desired disturbance attenuation factor and |*W*_3_(*jω*)| is the largest anticipated uncertainty of the plant expressed as a multiplicative perturbation. Note that the singular values of *S*(*jω*) determine the disturbance attenuation since *S*(*s*) is actually the closed-loop transfer function from an output disturbance *d* to plant output *y*. Note also that *T*(*s*) is indeed the closed-loop transfer function on the whole system. A stabilizing *H*_∞_ controller *K* is computed for plant *G* to make the sigma plot of the loop transfer function *GK* have desired loop shape *G*_*d*_ with accuracy *γ*. The specifications on disturbance attenuation and multiplicative stability margin in Eqs (22) and (23) can be written in terms of singular values of the loop transfer function since one can make the following approximation for $\bar{\sigma }\left(L\left(s\right)\right)\gg 1$

$$\begin{array}{c} S\left(s\right)={\left(I+L\left(s\right)\right)}^{-1}\approx L{\left(s\right)}^{-1}, \end{array}$$ (27)

and the one below for $\bar{\sigma }\left(L\left(s\right)\right)⪡1$

$$\begin{array}{c} T\left(s\right)=L\left(s\right){\left(I+L\left(s\right)\right)}^{-1}\approx L\left(s\right). \end{array}$$ (28)

Therefore if *ω*_*c*_ is the 0 dB crossover frequency of the singular values plot of *G*_*d*_(*jω*), the specifications can be stated as

$$\begin{array}{c} \underset{\bar{}}{\sigma }\left(G\left(j\omega \right)K\left(j\omega \right)\right)\geq \frac{1}{\gamma }\underset{\bar{}}{\sigma }\left(G_{d}\left(j\omega \right)\right),\;\forall \omega <\omega_{c} \end{array}$$ (29)

$$\begin{array}{c} \bar{\sigma }\left(G\left(j\omega \right)K\left(j\omega \right)\right)\leq \gamma \bar{\sigma }\left(G_{d}\left(j\omega \right)\right),\;\forall \omega >\omega_{c} \end{array}$$ (30)

Thus, high tracking performance is achieved at low frequencies where the system model is more accurate, and high robustness is achieved at high frequencies where the system model is less accurate and noise effects are stronger. A stable minimum-phase loop-shaping, squaring-down prefilter *W* is computed using greatest common divisor (GCD) formulas [50] such that the shaped plant *G*_*s*_ = *GW* is square and that the desired shape *G*_*d*_ is achieved with good accuracy in a desired frequency range (*ω*_*min*_, *ω*_*max*_) by the shaped plant; i.e.,

$$\begin{array}{c} \sigma \left(G_{d}\right)\approx \sigma \left(G_{s}\right),\forall \omega \in \left(\omega_{min},\omega_{max}\right). \end{array}$$ (31)

Normalized coprime factor synthesis theory is then used to compute an optimal loop-shaping controller for the shaped plant. If the shaped planet is factored as

$$\begin{array}{c} G_{s}=M^{-1}N \end{array}$$ (32)

then any perturbed plant can be written as

$$\begin{array}{c} G_{\Delta }={\left(M+\Delta_{M}\right)}^{-1}\left(N+\Delta_{N}\right) \end{array}$$ (33)

where Δ_*M*_ and Δ_*N*_ are stable and unknown transfer functions that represent uncertainties in the nominal plant. The objective of the robust controller design is to stabilize by a controller *K*, not only the nominal plant but also the family of perturbed plants defined as

$$G_{\varepsilon }={\left(M+\Delta_{M}\right)}^{-1}(N+\Delta_{N}):\Delta_{M},\Delta_{N}{}_{\infty }<\varepsilon .$$ (34)

For robust stability, internal stability must be achieved for the nominal and perturbed plant. If there exist a *K* such that (*M*, *N*, *K*, *ε*) is robustly stable, then (*M*, *N*, *ε*) is said to be robustly stabilizable with stability margin *ε* [51]. For robust stability the following must be satisfied

$$\begin{array}{c} {\left(I-GK\right)}^{-1},K{\left(I-GK\right)}^{-1},{\left(I-GK\right)}^{-1}G,\\ {\left(I-KG\right)}^{-1}\in RH_{\infty },\det\left(I-GK\right)\left(\infty \right)\neq 0\\ \underset{K}{\inf}{||\left[\begin{array}{c} (K{\left(I-GK\right)}^{-1}M^{-1}\\ {\left(I-GK\right)}^{-1}M^{-1}) \end{array}\right]||}_{\infty }\leq \varepsilon^{-1} \end{array}$$ (35)

where the infimum is taken over all stabilizing controllers. The *H*_∞_ optimization problem allows *ε*^−1^ being chosen as small as possible. For actual implementation, the robust stabilization problem can be converted to a more suitable formulation. Let

$$\begin{array}{c} P≐\left[\begin{array}{cc} P_{11} & P_{12}\\ P_{21} & P_{22} \end{array}\right]=\left[\begin{array}{cc} \left(\begin{array}{c} 0\\ M^{-1} \end{array}\right) & \left(\begin{array}{c} I\\ G \end{array}\right)\\ M^{-1} & G \end{array}\right] \end{array}$$ (36)

$$\begin{array}{c} F_{L}\left(P,K\right)≐P_{11}+P_{12}K{\left(I-P_{22}K\right)}^{-1}P_{21} \end{array}$$ (37)

Then (Eq 35) can be seen to be equivalent to

$$\begin{array}{c} \underset{K}{\inf}{∥F_{L}\left(P,K\right)∥}_{\infty }\leq \varepsilon^{-1} \end{array}$$ (38)

where *K* is gain chosen over all stabilizing controllers and *P* is a plant of standard form for *H*_∞_ optimization problem [52]. The final controller to be used is then computed as

$$\begin{array}{c} K_{final}=WK. \end{array}$$ (39)

### *μ*-synthesis using D-K Iteration

**Fig 2:**
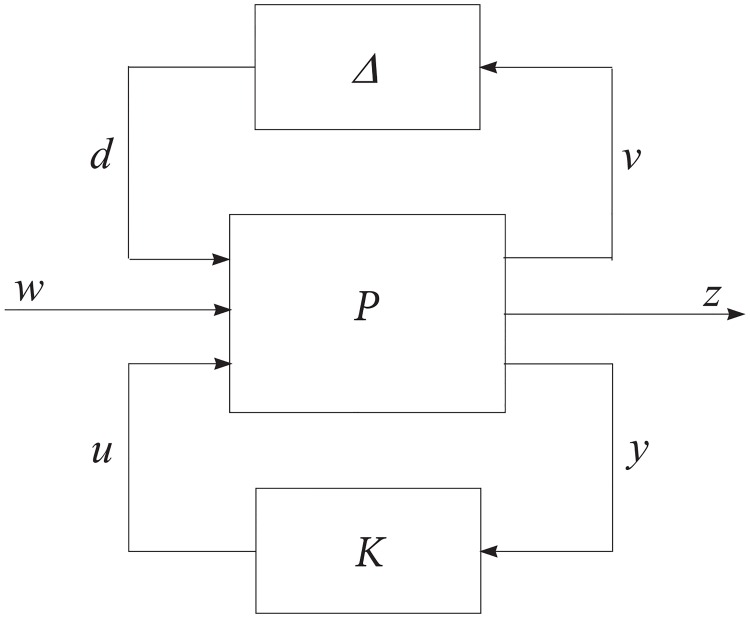
Control system configuration for *μ*-synthesis design.

Another MIMO control strategy utilized in this study is *μ*-synthesis, whose goal is to achieve robust performance in the presence of uncertainties. Consider the control system configuration in Fig 2. In the figure the nominal open-loop interconnected transfer function matrix is denoted *P*(*s*). This is the aircraft model without any uncertainties. The uncertainties in the parameters are represented by the transfer function matrix Δ(*s*) and the controller is denoted as *K*(*s*). The signals forming the interconnections are named *d*, *v*, *w*, *z*, *u* and *y* as shown in the figure. Based on these interconnections, *P*(*s*) can be partitioned as

$$\begin{array}{c} P(s)=\left[\begin{array}{ccc} P_{11} & P_{12} & P_{13}\\ P_{21} & P_{22} & P_{23}\\ P_{31} & P_{32} & P_{33} \end{array}\right]. \end{array}$$ (40)

The signals *y* are the feedback signals to the controller (i.e. tracking errors for our case) and *u* are the control signals generated by the controller. (i.e. the thrust and the surface deflections for our case.) The blocks *P*(*s*) and *K*(*s*) can be composed into a single block *M* as follows:

$$\begin{array}{l} M\left(P,K\right)=F_{l}\left(P,K\right)=\\ \;\left[\begin{array}{cc} P_{11} & P_{12}\\ P_{21} & P_{22} \end{array}\right]K{\left(I-P_{33}K\right)}^{-1}\left[\begin{array}{cc} P_{31} & P_{32} \end{array}\right]. \end{array}$$ (41)

The goal of *μ*-synthesis it to find a stabilising controller *K* such that

$$\begin{array}{c} \underset{\omega }{\sup}\mu \left[M\left(P,K\right)\left(j\omega \right)\right]<1 \end{array}$$ (42)

where *μ* is the structured singular value defined by

$$\begin{array}{c} \mu_{\Delta }\left(M\left(s\right)\right)=\underset{\omega }{\sup}\mu_{\Delta }\left(M\left(j\omega \right)\right) \end{array}$$ (43)

with

$$\begin{array}{c} \mu_{\Delta }^{-1}\left(M\left(j\omega \right)\right):=\min_{\Delta }\left\{\bar{\sigma }\left(\Delta \right):\det\left(I-M\left(j\omega \right)\Delta \right)=0\right\}. \end{array}$$

This is usually expressed as the optimization problem

$$\begin{array}{c} \underset{K(s)}{\inf}\underset{\omega }{\sup}\mu \left[M\left(P,K\right)\left(j\omega \right)\right]. \end{array}$$ (44)

An iterative approach was introduced in [53] to solve (Eq 44). The method is called the D-K iteration *μ*-synthesis method, and is based on solving the following optimization problem, for a stabilising controller *K* and a diagonal constant scaling matrix *D*,

$$\begin{array}{c} \underset{K(s)}{\inf}\underset{\omega }{\sup}\underset{D}{\inf}\bar{\sigma }\left[DM\left(P,K\right)D^{-1}\left(j\omega \right)\right]. \end{array}$$ (45)

Referring to (Eq 42), a stabilising controller is to be found such that

$$\begin{array}{c} \underset{\omega }{\sup}\underset{D}{\inf}\bar{\sigma }\left[DM\left(P,K\right)D^{-1}\left(j\omega \right)\right]<1. \end{array}$$ (46)

The D-K iteration method minimizes (Eq 45), i.e. reduces the left-hand-side value of (Eq 46) for *K* and *D* in turn, while keeping the other one fixed. For a given matrix *D*, (Eq 45) is a standard *H*_∞_ optimisation problem

$$\begin{array}{c} \underset{K(s)}{\inf}{∥DM\left(P,K\right)D^{-1}∥}_{\infty } \end{array}$$ (47)

that can be written as

$$\begin{array}{c} \underset{K}{\inf}∥DF_{l}\left(P,K\right)D^{-1}{∥}_{\infty }=\underset{K}{\inf}{∥F_{l}\left(\tilde{P},K\right)∥}_{\infty } \end{array}$$ (48)

with

$$\begin{array}{c} \tilde{P}=\left[\begin{array}{cc} D & 0\\ 0 & I \end{array}\right]P\left[\begin{array}{cc} D^{-1} & 0\\ 0 & I \end{array}\right] \end{array}$$ (49)

compatible with the partition of *P*. For a fixed *K*(*s*), ${\text{inf}}_{D}\;\bar{\sigma }\left[DM\left(P,K\right)D^{-1}\left(j\omega \right)\right]$ is a convex optimization problem at each frequency *ω*. After minimization on a frequency range of interest, the resultant diagonal matrices can be approximated using curve fitting, by a stable minimum phase, rational transfer function matrix *D*(*s*). This is then used in the next iteration for *K*. The steps of the D-K iterative *μ*-synthesis algorithm can be summarized as follows:

1. Start with an initial guess for *D*, usually *D* = *I*.
2. Fix *D* and solve the *H*_∞_-optimization for *K*,
  $$\begin{array}{c} K=\arg\underset{K}{\inf}{∥F_{l}\left(\tilde{P},K\right)∥}_{\infty }. \end{array}$$ (50)
3. Fix *K* and solve the following convex optimization problem for *D* at each frequency over a desired frequency range,
  $$\begin{array}{c} D\left(j\omega \right)=\arg\underset{D}{\inf}\bar{\sigma }\left[DF_{l}\left(P,K\right)D^{-1}\left(j\omega \right)\right]. \end{array}$$ (51)
4. Curve fit *D*(*jω*) to get a stable, minimum-phase *D*(*s*). Go to Step 2 and repeat, until a desired convergence tolerance is met, (Eq 46) is achieved, or a prespecified maximum iteration count is reached.

It is well known that the solution to the H-infinity optimization problem (Eq 50) is not unique except in the scalar case and there are no analytic formulae for the solutions in general [54]. In practice one usually seeks a suboptimal solution close enough to the actual one, i.e. one tries to find a controller *K* such that

$$\begin{array}{c} ∥F_{l}\left(\tilde{P},K\right){∥}_{\infty }<\gamma \end{array}$$ (52)

for a small enough value of *γ* > 0. Several established methods exist for solving (Eq 52) among which we utilize the two-Riccati formulae, the mathematical details of which can be found in [55]. A slight extension is employed here in the sense that multiple iterations are performed with successively smaller values of *γ*. Starting with a conservative (high) and tight (low) bound guess for *γ*, a bisection algorithm is applied to approach the optimal *γ* value. At each step, the problem (Eq 52) may or may not be feasible depending on how small *γ* is. The algorithm terminates and returns the last feasible solution obtained when the relative difference between the last *γ* value that failed and the last *γ* value that succeeded is less than a specified tolerance (0.01 for this work).

The D-K iteration approach used in this study is implemented using the numerical computing package MATLAB. It will be employed in the succeeding sections to design a MIMO controller for the aircraft model with a prescribed amount of parameter variation in an attempt to achieve robust performance over the entire uncertainty range.

*Remark*. At this point it worth recapping why loop-shaping and *μ*-synthesis were chosen as the control design methods above others. While various tools exists for building robust controllers, loop-shaping and *μ*-synthesis were preferred since their design procedures can be linked (directly or indirectly) to the control of MIMO systems under parametric variations. In loop-shaping, the procedure is based on finding a controller to make the loop transfer function match a desired loop shape. The properties of the desired loop shape such as low/high frequency gains, bandwidth and crossover slope can be used to specify performance and robustness margins. These will in turn determine the behaviour under undesired circumstances including parameter uncertainties. As to the *μ*-synthesis based on D-K iteration technique, this is a numerical method where the amount of anticipated uncertainty can be specified directly as a constraint for optimization. Once the procedure converges to a solution, the resulting controller is guaranteed work for the modelled uncertainty.

### Blade Element Simulation

**Fig 3:**
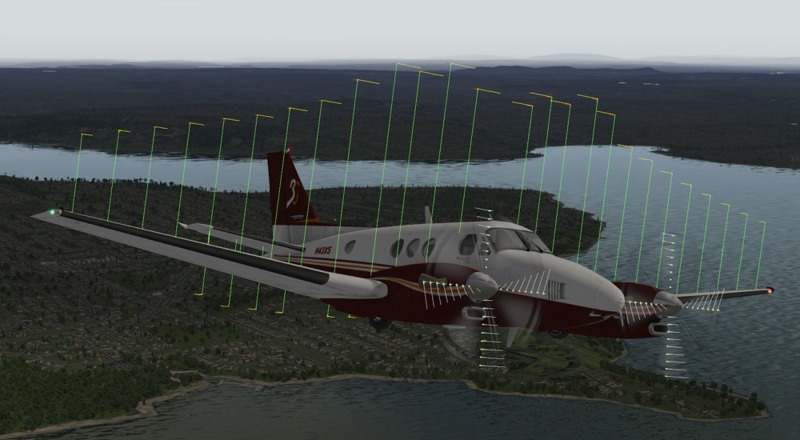
During flight simulation, the aircraft is split into a number of surfaces and the forces on each are computed by BET. Reprinted from http://www.x-plane.com/desktop/how-x-plane-works/ under a CC BY 4.0, with permission from Laminar Research, original copyright 2011.

**Fig 4:**
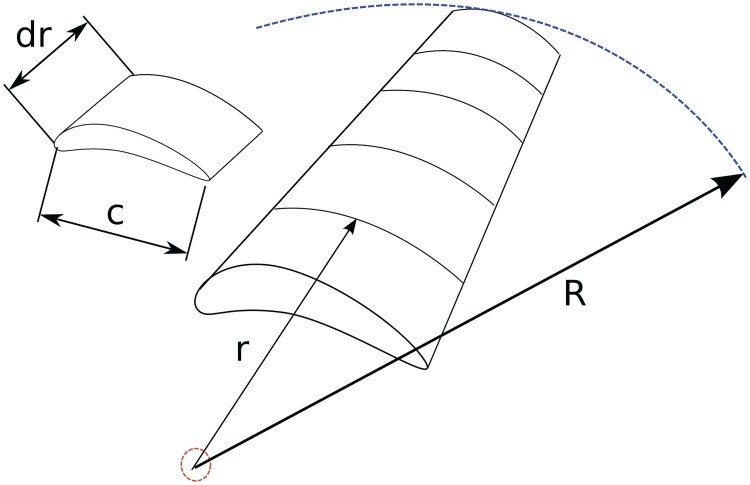
Illustration of the main idea of blade element theory (BET) on a propeller blade.

After nonlinear dynamical simulations, the final test for the control system is software-in-the-loop (SIL) verifications based on blade element theory (BET). The surfaces of the aircraft (e.g. propellers, wings, stabilizers) are divided into several sections, the lift/drag forces acting on each section is computed separately and the composite effect is applied to the entire aerial vehicle (Fig 3). This approach contrasts traditional flight simulations relying on empirical data (e.g. stability derivatives) in predefined lookup tables and is widely accepted to be more realistic albeit computationally expensive. It is also a good choice for testing the control design presented here as the mathematical model utilized is based on stability derivatives. Hence a different (and more accurate) flight simulation technique that does not rely on stability derivatives serves as a better test. The main idea of BET can be summarized on a propeller blade shown in Fig 4. The blade is divided into *N* elements, each of which experiences a slightly different flow. Lift and drag coefficients (*C*_*L*_, *C*_*D*_) are readily available for numerous airfoil shapes from wind tunnel tests. Using relative velocities, the flow over each element can be related to these tests. The flow is slightly turned passing over the airfoil so inlet and exit flow conditions are averaged to improve accuracy. Carrying out the necessary computations yields

$$\begin{array}{c} dF_{x}=dL\sin\beta +dD\cos\beta \end{array}$$ (53)

$$\begin{array}{c} dF=dL\text{cos}\beta -dD\text{sin}\beta \end{array}$$ (54)

$$\begin{array}{c} dL=\sigma^{\prime }\pi \rho \frac{V^{2}{\left(1-a\right)}^{2}}{\cos^{2}2\beta }C_{L}rdr \end{array}$$ (55)

$$\begin{array}{c} dD=\sigma^{\prime }\pi \rho \frac{V^{2}{\left(1-a\right)}^{2}}{\cos^{2}2\beta }C_{D}rdr \end{array}$$ (56)

$$\begin{array}{c} \sigma^{\prime }=\frac{Bc}{2\pi r} \end{array}$$ (57)

where *dL*, *dD*, *dF*_*x*_, *dF*_*θ*_ are respectively the lift, drag, axial and tangential forces, *β* is the relative flow angle, *ρ* is the air density, *V* is the flow velocity, *a* is the axial induction factor, *r* is the radius, *σ*′ is the local solidity, B is the number of blades and c is chord length [41]. The procedure is carried out on the entire aircraft to compute all the forces, using which the flight dynamics can be simulated.

## Results

**Fig 5:**
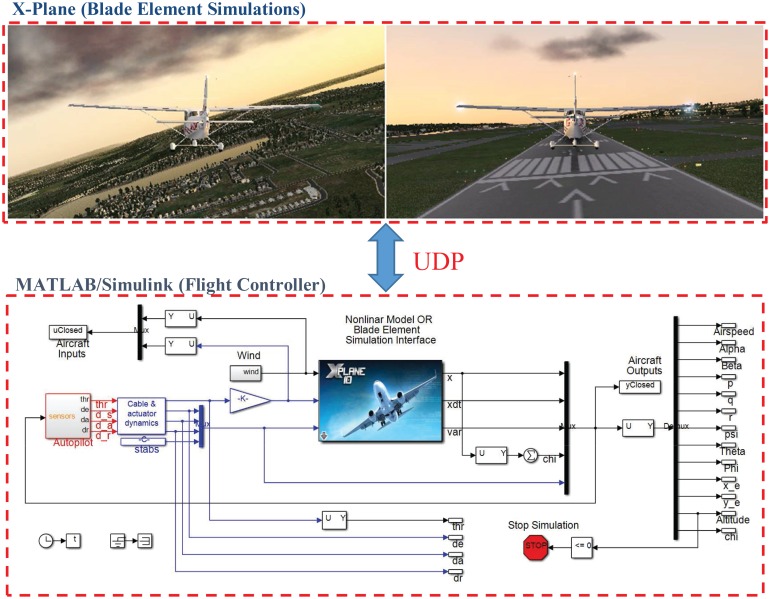
The setup for verification of the results through nonlinear dynamical simulations and/or SIL tests based on BET.

In this part first the issues linked to the conventional independent SISO control strategy is highlighted on the attitude control (inner loop) of a popular general aviation aircraft, namely the Cessna 172. The mass, geometry parameters, performance data and stability derivatives of this aircraft are given in Tables 1 and 2. The objective is to use the four usual inputs, namely, thrust *F*_*X*_, elevator deflection *δ*_*e*_, aileron deflection *δ*_*a*_ and rudder deflection *δ*_*r*_ to manipulate the four outputs airspeed *V*, pitch angle *θ*, roll angle *ϕ* and sideslip angle *β*. The outcomes of the are conventional individual-channel SISO approach are compared to the MIMO control design methods loop-shaping and *μ*-synthesis, which drastically enhance the robustness to parameter changes. Next the functionality of the robust MIMO control approach is additionally validated via the inclusion of an outer loop for path following. For tests the flight controller is first coded in MATLAB/Simulink and simulated on the nonlinear aircraft equations given in Section Mathematical Model. Ultimately SIL simulations are carried out by interfacing the flight controller block with blade element simulations performed by the flight simulator X-Plane. The former transmits thrust, elevator, aileron, rudder commands to the latter and receives flight simulation data in real-time every 25 ms by means of user datagram protocol (UDP) packets. The setup is shown in Fig 5.

**Table 1:** Mass, geometry parameters and performance specifications for Cessna 172.

Parameter	Value
$\bar{c}$	1.4935 m
*b*	10.9118 m
*S*	16.1651 m^2^
*I*_*xx*_	1285.3 kg.m^2^
*I*_*yy*_	1824.9 kg.m^2^
*I*_*zz*_	2666.9 kg.m^2^
*J*_*xy*_	0 kg.m^2^
*J*_*xz*_	0 kg.m^2^
*J*_*yz*_	0 kg.m^2^
*m*	1043.3 kg
Cruise speed	65 m/s
Stall speed	24 m/s
Never exceed speed	84 m/s
Maximum crosswind	7.7 m/s
Service ceiling	4100 m

**Table 2:** Stability derivatives for Cessna 172.

Derivative	Value
*C*_*D*_0__	0.031
*C*_*D*_*α*__	0.13
*C*_*D*_*q*__	0
$C_{D_{\delta_{e}}}$	0.06
$C_{D_{j_{h}}}$	0
*C*_*L*_0__	0.31
*C*_*L*_*α*__	5.143
*C*_*L*_*q*__	3.9
$C_{L_{\delta_{e}}}$	0.43
$C_{L_{j_{h}}}$	0
*C*_*Y*_*β*__	-0.31
*C*_*Y*_*p*__	-0.037
*C*_*Y*_*r*__	0.21
$C_{Y_{\delta_{a}}}$	0.0
$C_{Y_{\delta_{r}}}$	0.187
*C*_*l*_0__	0
*C*_*l*_*β*__	-0.089
*C*_*l*_*p*__	-0.47
*C*_*l*_*r*__	0.096
$C_{l_{\delta_{a}}}$	-0.178
$C_{l_{\delta_{r}}}$	0.0147
*C*_*m*_0__	-0.015
*C*_*m*_*α*__	-0.89
*C*_*m*_*q*__	-12.4
$C_{m_{\delta_{e}}}$	-1.28
$C_{m_{j_{h}}}$	0
*C*_*n*_0__	0
*C*_*n*_*β*__	0.065
*C*_*n*_*p*__	-0.03
*C*_*n*_*r*__	-0.099
$C_{n_{\delta_{a}}}$	-0.053
$C_{n_{\delta_{r}}}$	-0.0657

### Independent SISO Control Design

We consider a typical flight condition for the Cessna 172, namely straight and level flight at *V*_0_ = 65 m/s and elevation *z*_*e*, 0_ = 1000 m. A trim point for this condition is computed as follows

$$\begin{array}{l} x_{0}=\left[v,\alpha ,\beta ,p,q,r,\psi ,\theta ,\phi ,x_{e},y_{e},z_{e}\right]\\ \;=\left[65,-0.00729,0,0,0,0,0,-0.00729,0,0,0,1000\right]\\ u_{0}=\left[F_{x},\delta_{e},\delta_{a},\delta_{r}\right]=\left[1125.7,-0.00665,0,0\right]. \end{array}$$

**Fig 6:**
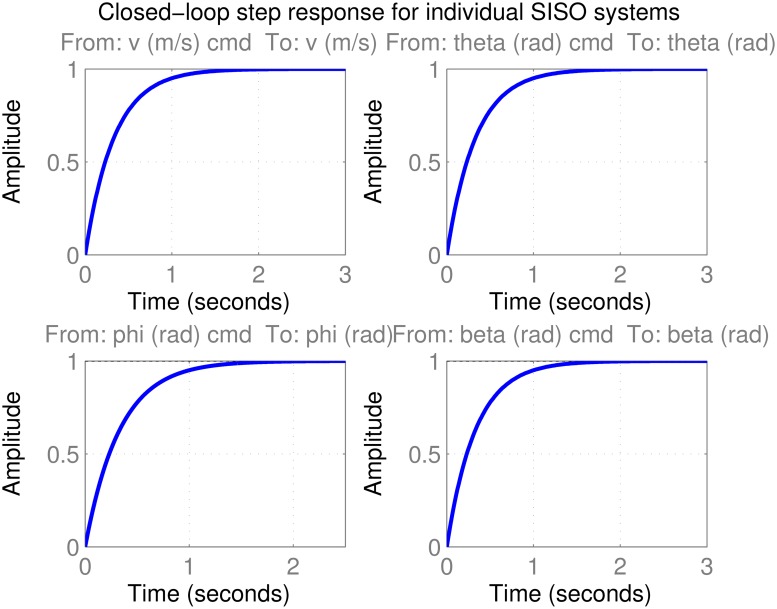
Closed-loop step response for individual SISO systems.

**Fig 7:**
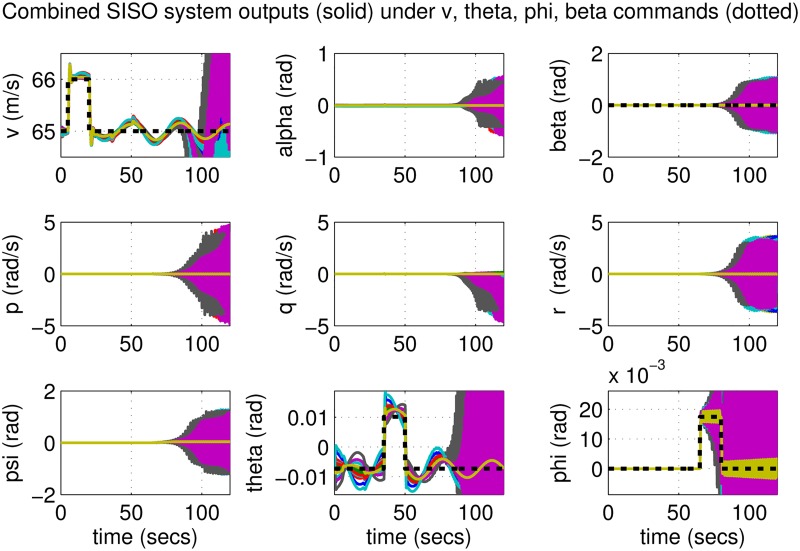
States of perturbed aircrafts under combined SISO control.

Defining the output as

$$\begin{array}{c} y={\left[V\;\theta \;\phi \;\beta \right]}^{T} \end{array}$$ (58)

a linearization around (*x*_0_, *u*_0_) yields a 4 × 4 transfer function matrix

$$\begin{array}{c} G\left(s\right)=\left[\begin{array}{cccc} G_{11}\left(s\right) & G_{12}\left(s\right) & G_{13}\left(s\right) & G_{14}\left(s\right)\\ G_{21}\left(s\right) & G_{22}\left(s\right) & G_{23}\left(s\right) & G_{24}\left(s\right)\\ G_{31}\left(s\right) & G_{32}\left(s\right) & G_{33}\left(s\right) & G_{34}\left(s\right)\\ G_{41}\left(s\right) & G_{42}\left(s\right) & G_{43}\left(s\right) & G_{44}\left(s\right) \end{array}\right] \end{array}$$ (59)

representing the local behavior around *V*_0_ = 65 m/s and *z*_*e*, 0_ = 1000 m. For individual SISO design, since there are four inputs, one can select four of these *G*_*ij*_(*s*) and design separate controllers. It makes sense to pick the diagonal entries *G*_11_, *G*_22_, *G*_33_, *G*_44_ since for straight and level flight, *V* is mostly influenced by *F*_*x*_, *θ* by *δ*_*e*_, *ϕ* by *δ*_*a*_ and *β* by *δ*_*r*_. For a different scenario an alternate choice may be preferable. For instance during landing many pilots think of regulating airspeed by elevator, altitude by thrust, keeping the aircraft level with ailerons, and aligning with the runway using the rudder. A standard SISO control design is then performed on each diagonal entry, hoping that the off-diagonal dynamics remain well-behaved in closed-loop. To design the individual controllers, several standard automated tuning methods such as Ziegler-Nichols PID, internal model control (IMC), linear quadratic Gaussian (LQG) and optimization based approaches were tested and the best results were obtained for the IMC design method [56, 57] with a time constant of *τ* = 1/3 s ≈ 0.3333 s, resulting in well-damped responses settling in about *t_s_* = 4τ ≈ 1.3333 s as seen in Fig 6. While the controllers seem to perform well on their individual nominal models, the real test is whether they will regulate the aircraft attitude successfully when used simultaneously under parameter uncertainties in nonlinear simulations. A sample scenario is presented in Fig 7 where the aircraft is commanded to do the following:

1. Increase airspeed *V* by 1 m/s at *t* = 5 s and decrease it by the same amount at *t* = 20 s.
2. Increase pitch angle *θ* by 1 degree (0.0175 rad) at *t* = 35 s and decrease it by the same amount at *t* = 50 s.
3. Increase roll angle *ϕ* by 1 degree (0.0175 rad) at *t* = 65 s and decrease it by the same amount at *t* = 80 s.
4. Keep sideslip angle *β* at zero for all times.

One hundred Monte-Carlo simulations on the nonlinear model were performed, allowing up to a mere 2% perturbation in each aircraft parameter. The resulting aircraft states are plotted in Fig 7. It can be seen that some of the responses provide acceptable tracking of the references; these are for parameters close to nominal values listed in Table 1. However, it is clear from the figure that certain parameter combinations have poor performance with significant oscillations, some of which even grow unboundedly. In practice this would imply the destabilization and potential loss of the aircraft. It is true that these scenarios are unlikely and constitute a small portion of all the runs. However any risk of losing an aircraft and the lives of those onboard justifies the need to consider alternate autopilot strategies. One such strategy is investigated in the next section.

### Loop-shaping MIMO Control Design

For loop-shaping control design the system is first linearized around the nominal flight condition *V*_0_ = 65 m/s and *z*_*e*, 0_ = 1000 m for the parameters given in Tables 1 and 2. The procedure is identical to that described in previous section until (Eq 59). Simple actuator dynamics are also augmented to *G*(*s*) for a more realistic model, i.e.

$$\begin{array}{c} G_{nom}\left(s\right)=G\left(s\right)G_{act}\left(s\right) \end{array}$$ (60)

where

$$\begin{array}{c} G_{act}\left(s\right)=\text{diag}\left(\frac{4}{s+4},\frac{15}{s+15},\frac{40}{s+40},\frac{15}{s+15}\right). \end{array}$$ (61)

and diag stands for diagonal matrix. *G*_*act*_ captures the fact that the real Cessna 172 reacts slower to throttle command, somewhat faster to elevator and rudder commands, and the fastest to aileron commands. Therefore the controller must not generate commands beyond these bandwidth or else they will not be effective. The loop-shaping is controller designed following the procedure in Section *H*_∞_ Loop-shaping with desired loop-shape

$$\begin{array}{c} G_{d}\left(s\right)=\text{diag}\left(\frac{3}{s},\frac{3}{s},\frac{3}{s},\frac{3}{s}\right). \end{array}$$ (62)

**Fig 8:**
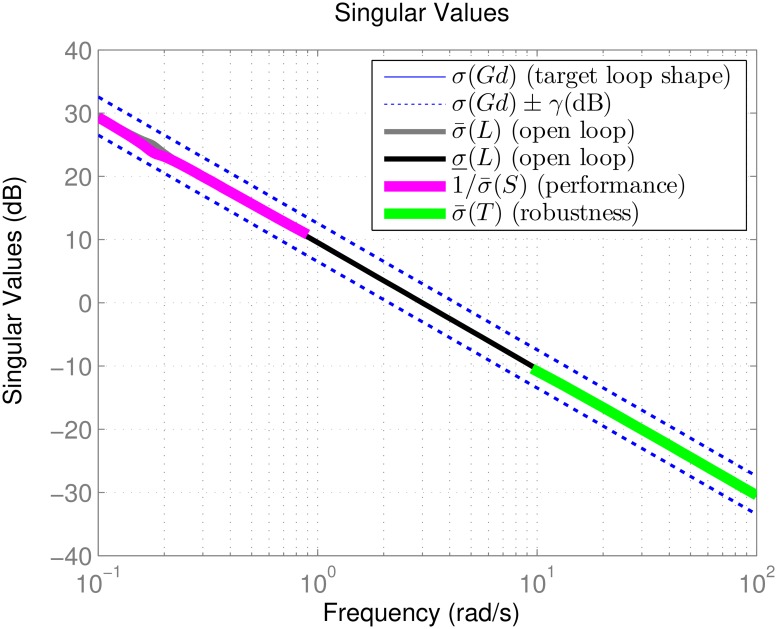
Singular value plot for the loop-shaping controller.

Fig 8 shows the singular value plot, where the loop transfer function *L*(*s*) is seen to approximate *G*_*d*_(*s*) within tolerance bound *γ* = 1.4155. The bounds on the singular values of the sensitivity function *S*(*s*) and complementary sensitivity function *T*(*s*) favor performance at low frequencies and robustness at high frequencies, as described in Section *H*_∞_ Loop-shaping. Since *L*(*s*) ≈ *G*_*d*_(*s*), this suggests that

$$\begin{array}{c} T\left(s\right)=L\left(s\right){\left(I+L\left(s\right)\right)}^{-1}\approx G_{d}\left(s\right){\left(I+G_{d}\left(s\right)\right)}^{-1} \end{array}$$ (63)

from where one obtains

$$\begin{array}{c} T(s)\approx \text{diag}\left(\frac{3}{s+3},\frac{3}{s+3},\frac{3}{s+3},\frac{3}{s+3}\right)\\ =\left[\begin{array}{cccc} \frac{3}{s+3} & 0 & 0 & 0\\ 0 & \frac{3}{s+3} & 0 & 0\\ 0 & 0 & \frac{3}{s+3} & 0\\ 0 & 0 & 0 & \frac{3}{s+3} \end{array}\right] \end{array}$$ (64)

From here one observes that the individual transfer functions for the diagonal channels are approximated by

$$\begin{array}{c} \frac{V_{cmd}\left(s\right)}{V(s)}=\frac{\theta_{cmd}\left(s\right)}{\theta (s)}=\frac{\phi_{cmd}\left(s\right)}{\phi (s)}=\frac{\beta_{cmd}\left(s\right)}{\beta (s)}\approx \frac{3}{s+3} \end{array}$$ (65)

so the closed loop system will be able to track all references successfully with minimal overshoot and a settling time of approximately $t_{s}=5\tau =5\frac{1}{2}=2.5$ seconds. Also the off-diagonal entries of *T*(*s*) are roughly zero, which indicates that the coupling between different command-response pairs are eliminated.

**Fig 9:**
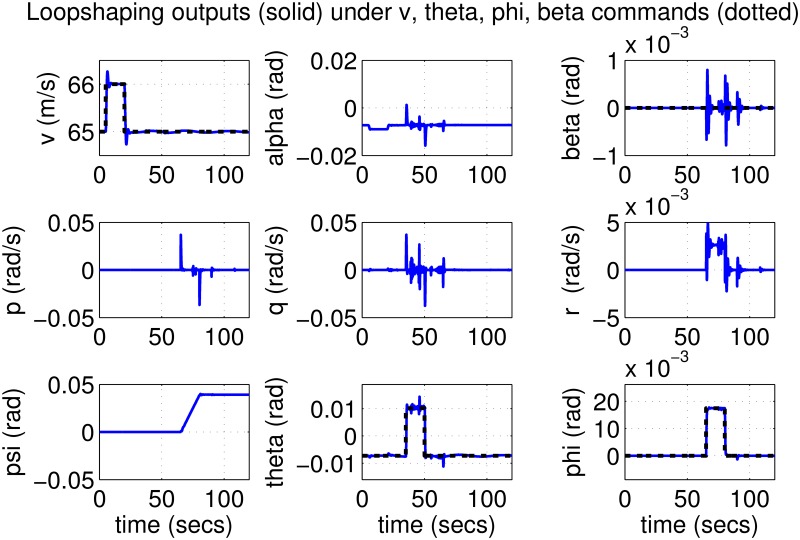
States of the nominal aircraft under loop-shaping control.

**Fig 10:**
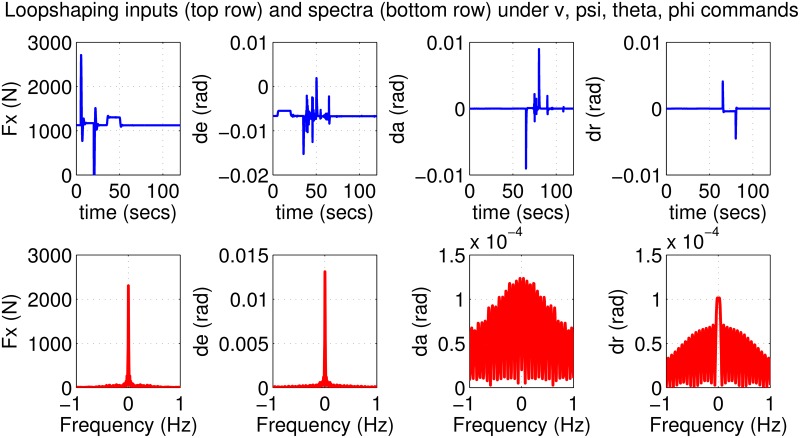
Inputs to the nominal aircraft under loop-shaping control.

**Fig 11:**
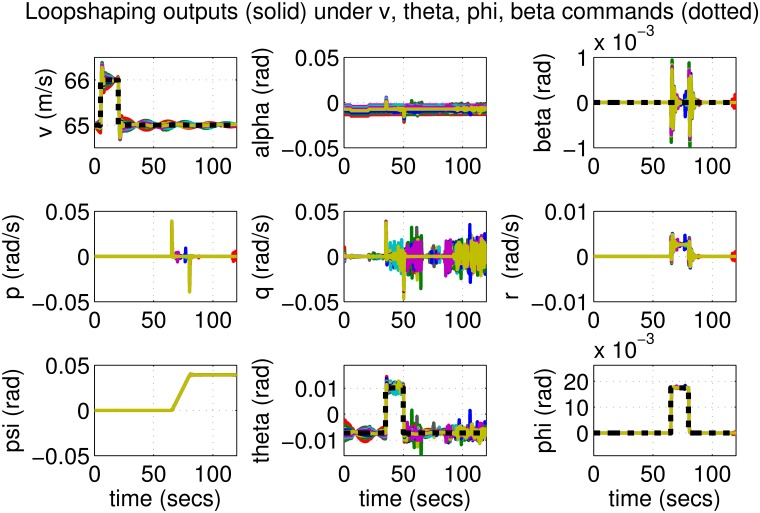
States of perturbed aircrafts under loop-shaping control.

**Fig 12:**
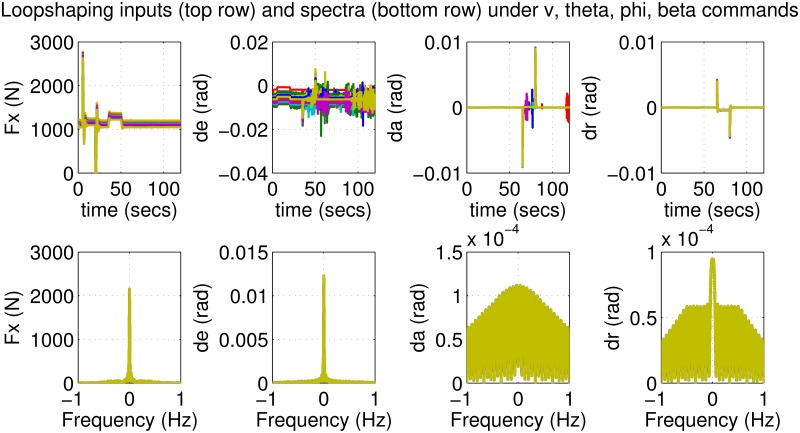
Inputs of perturbed aircrafts under loop-shaping control.

The results for the loop-shaping controller in closed-loop for the nonlinear aircraft with the nominal parameter values are shown in Fig 9. The nominal performance is seen to be quite good with accurate tracking and very little cross-coupling. The inputs generated by the controller are also shown in Fig 10. The thrust and surface deflections remain within reasonable limits at all times and the control inputs do not contain significant power at frequencies higher than about 0.5 Hz. This ensures that the control does not cause any sharp thrust changes or wild oscillations in control surfaces. The responses of the loop-shaping controller to perturbed nonlinear models are shown in Fig 11. One hundred Monte-Carlo simulations on the nonlinear model were performed, allowing up to 20% perturbation in each aircraft parameter. It is seen that the stability is never lost, and acceptable tracking performance is achieved for all cases. The inputs to the aircraft are also plotted in Fig 12 showing that the inputs always remain within reasonable amplitude and frequency ranges.

### *μ*-synthesis MIMO Control Design

For *μ*-synthesis control design the system must be expressed in the form described in Section *μ*-synthesis using D-K Iteration. For this purpose, the nominal aircraft model *G*_*nom*_(*s*) is defined based on *G*(*s*) in (Eq 59), i.e. the linearization around *V*_0_ = 65 m/s and *z*_*e*, 0_ = 1000 m for the parameters given in Tables 1 and 2. We also augment simple actuator dynamics for a more realistic model, i.e.

$$\begin{array}{c} G_{nom}\left(s\right)=G\left(s\right)G_{act}\left(s\right) \end{array}$$ (66)

where

$$\begin{array}{c} G_{act}\left(s\right)=\text{diag}\left(\frac{4}{s+4},\frac{15}{s+15},\frac{40}{s+40},\frac{15}{s+15}\right). \end{array}$$ (67)

and diag stands for diagonal matrix. *G*_*act*_ captures the fact that the real Cessna 172 reacts slower to throttle command, somewhat faster to elevator and rudder commands, and the fastest to aileron commands. Therefore the controller must not generate commands beyond these bandwidth or else they will not be effective. Parameter variations are expressed as an input multiplicative uncertainty Δ_*M*_(*s*) so that the real aircraft model is

$$\begin{array}{c} G_{real}=G_{nom}\left(s\right)\left(I+\Delta_{M}\left(s\right)\right). \end{array}$$ (68)

**Fig 13:**
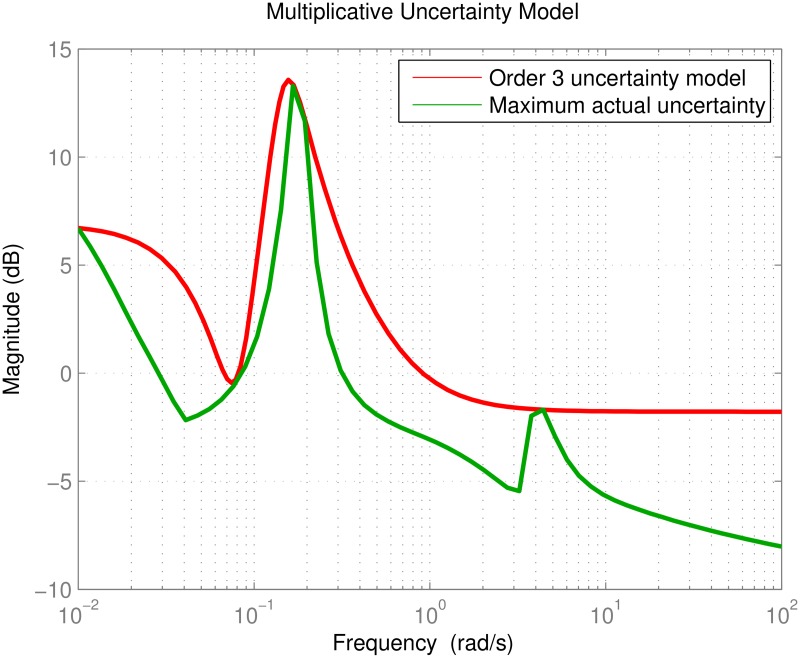
Third order multiplicative uncertainty model.

**Fig 14:**
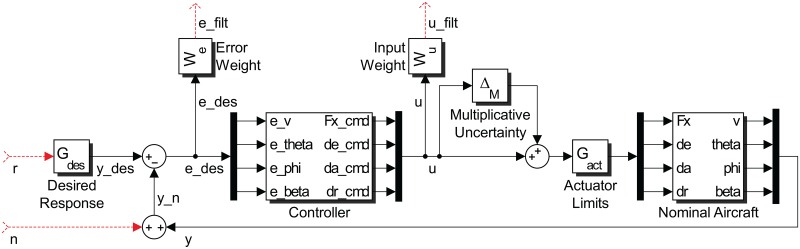
Block diagram for *μ*-synthesis control design via D-K iteration.

The goal is set to design a controller that can tolerate up to 20% uncertainty on the aircraft parameters; this figure is consistent with the typical variation in parameter values estimated by AAA, AVL, wind-tunnel tests and actual flight data as described in the Introduction. To compute Δ_*M*_(*s*) the parameters are randomly perturbed up to 20%, a linearized model is obtained for each perturbed parameter set, and the procedure is repeated 100 times to collect enough data. The variations between the nominal model *G*_*nom*_ and all 100 models are computed and an uncertainty Δ_*M*_(*s*) is selected to cover the maximum variation over the frequency range of interest, which is chosen as *ω* ∈ (0.01, 100) rad/s for this application. Due to the large data set, it would be time consuming and computationally intensive to estimate individual covers for all 16 channels so we utilize a scalar third order Δ_*M*_(*s*) to cover the uncertainties over all channels:

$$\begin{array}{c} \Delta_{M}\left(s\right)=\frac{0.8145s^{3}+0.5402s^{2}+0.02681s+0.003253}{s^{3}+0.1437s^{2}+0.0275s+0.00147} \end{array}$$

which is seen from Fig 13 to successfully bound the uncertainty within the frequency range of interest. With Δ_*M*_(*s*) at hand, the block diagram in Fig 14 is constructed to carry out *μ*-synthesis control design. The main goal is to match the response of the system to a desired response contained in the block *G*_*des*_(*s*), i.e. to minimize the error *e*_*des*_. Similar to the independent SISO design case, we ask for the diagonal channels to be well damped with a time constant of *τ* = 1/3 s = 0.333 s. Unlike the independent SISO design however, we have the opportunity to specify the desired response for the off-diagonal entries, which would ideally be zero for perfect decoupling. In summary *G*_*des*_(*s*) is picked as

$$\begin{array}{c} G_{des}\left(s\right)=\left[\begin{array}{cccc} \frac{3}{s+3} & 0 & 0 & 0\\ 0 & \frac{3}{s+3} & 0 & 0\\ 0 & 0 & \frac{3}{s+3} & 0\\ 0 & 0 & 0 & \frac{3}{s+3} \end{array}\right]. \end{array}$$ (69)

*W*_*e*_ and *W*_*u*_ are weighting filters for the error and inputs to select certain frequencies during optimization for *μ*-synthesis. It is natural to emphasize low frequencies for tracking purposes so a first order filter with DC-gain 50, bandwidth 3 rad/s, and high frequency gain 0.02 is selected

$$\begin{array}{c} W_{e}=\frac{0.02s+3}{s+0.06}. \end{array}$$ (70)

The inputs generated by the controller must adhere to the frequency limitations of the actuators so we pick *W*_*u*_ = *G*_*act*_ to represent this criterion.

Establishing correspondence between Figs 14 and 2, Δ is Δ_*M*_, *K* is the block labeled “Controller” and *P* contains everything else. The external inputs are *w* = [*r* *n*]^*T*^, where *r* are the reference commands, and *n* is an output disturbance representing sensor noise, atmospheric effects and so on. The overall outputs of the system are *z* = [*e*_*filt*_ *u*_*filt*_]^*T*^, which are the weighted error and weighted input signals. The system is therefore in suitable for for D-K iteration described in Section *μ*-synthesis using D-K Iteration so the procedure is executed numerically using MATLAB to design the controller. The procedure returned a *K* satisfying sup_*ω*_ *μ*[*M*(*P*, *K*)(*jω*)] < 0.9174 indicating a successful design in accordance with (Eq 42).

**Fig 15:**
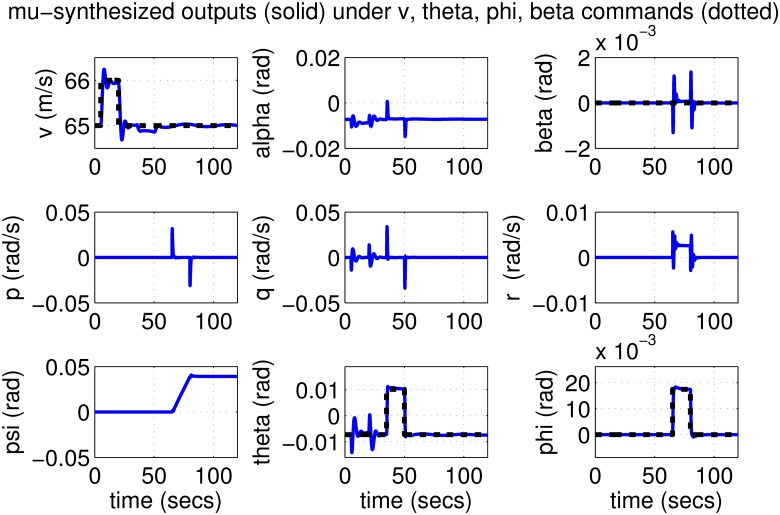
States of the nominal aircraft under *μ*-synthesis control.

**Fig 16:**
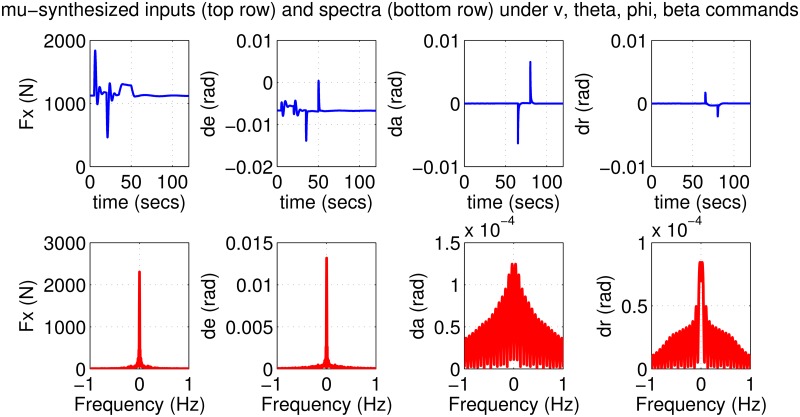
Inputs to the nominal aircraft under *μ*-synthesis control.

**Fig 17:**
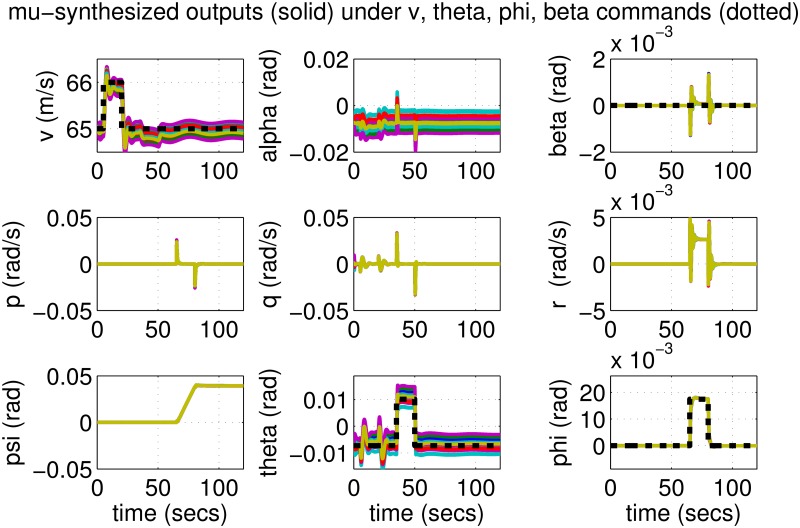
States of perturbed aircrafts under *μ*-synthesis control.

**Fig 18:**
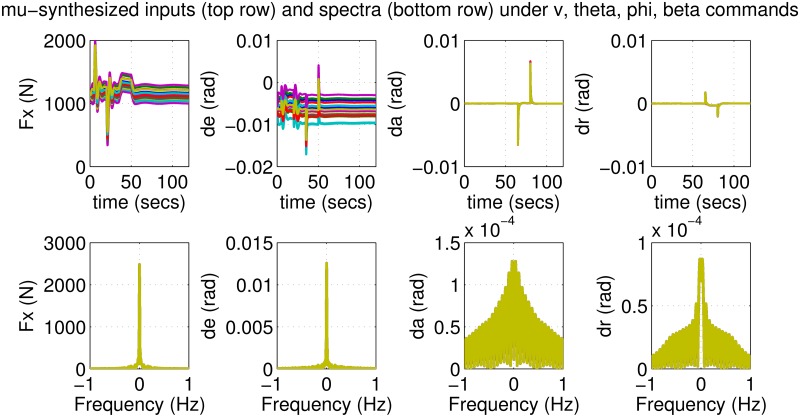
Inputs of perturbed aircrafts under *μ*-synthesis control.

The results for the *μ*-synthesized controller in closed-loop for the nonlinear aircraft with the nominal parameter values is shown in Fig 15. The nominal performance is seen to be quite good with accurate tracking and very little cross-coupling. The inputs generated by the controller are also shown in Fig 16. The thrust and surface deflections remain within reasonable limits at all times and the control inputs do not contain significant power at frequencies higher than about 0.5 Hz. This ensures that the control does not cause any sharp thrust changes or wild oscillations in control surfaces. The responses of the *μ*-synthesized controller to perturbed nonlinear models are shown in Fig 17. One hundred Monte-Carlo simulations on the nonlinear model were performed, allowing up to 20% perturbation in each aircraft parameter. It is seen that the stability is never lost, and acceptable tracking performance is achieved for all cases. The inputs to the aircraft are also plotted in Fig 18 showing that the inputs always remain within reasonable amplitude and frequency ranges.

*Remark*. While the results above confirm robust performance and stability to the designed amount of uncertainty (20%), the controller in practice is able to handle much higher perturbations. We performed over 1000 simulations where we have seen that the performance remains acceptable to around 34%, and stability is not lost up to around 43% parameter variation. This is consistent with the knowledge that *μ*-synthesis control design via D-K iteration can be suboptimal and produce conservative results.

### Outer Loop and Blade Element Simulation Tests

As a final test, an outer-loop controller is wrapped around the attitude controller to generate attitude references (*r* in Fig 14) from outer-loop references *r*_*outer*_ = [*r*_*v*_ *r*_*z*_*e*__ *r*_*χ*_ *r*_*β*_]^*T*^. These are the airspeed command, altitude command, heading angle command and sideslip angle command respectively. The outer and inner loop collectively form a complete flight control system capable of navigation and landing. In this section the results are presented for both loops designed with loop-shaping but similar outcomes were also obtained using *μ*-synthesis.

**Fig 19:**
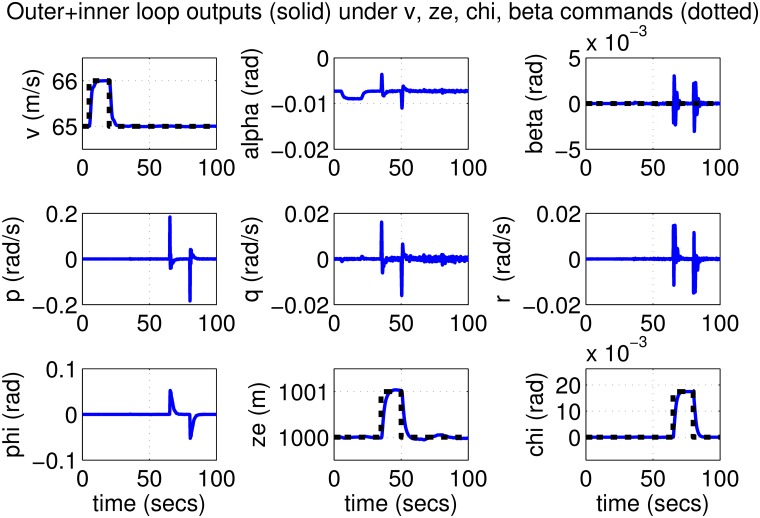
States of the nominal aircraft with loop-shaped inner and outer controllers.

**Fig 20:**
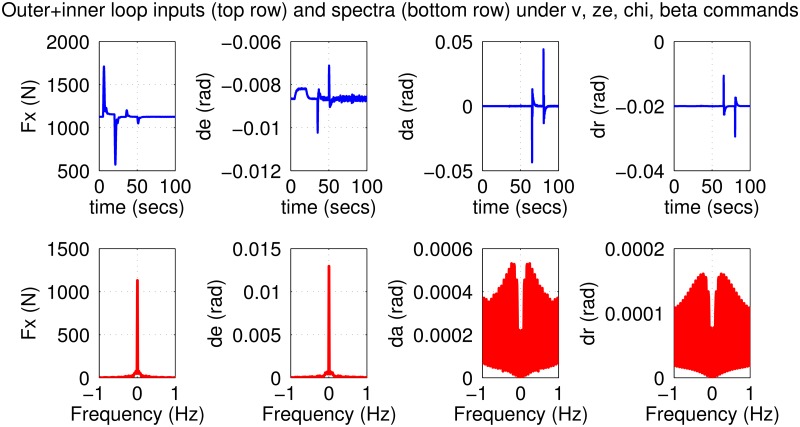
Inputs to the nominal aircraft with loop-shaped inner and outer controllers.

**Fig 21:**
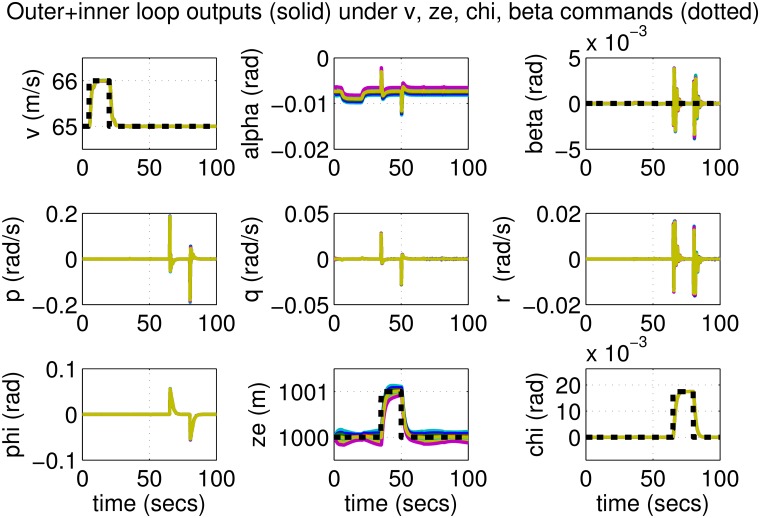
Outputs of perturbed aircrafts with loop-shaped inner and outer controllers.

**Fig 22:**
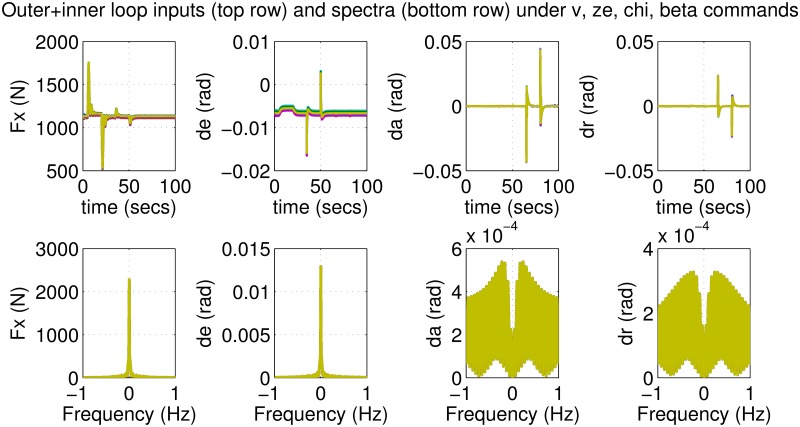
Inputs to perturbed aircrafts with loop-shaped inner and outer controllers.

**Fig 23:**
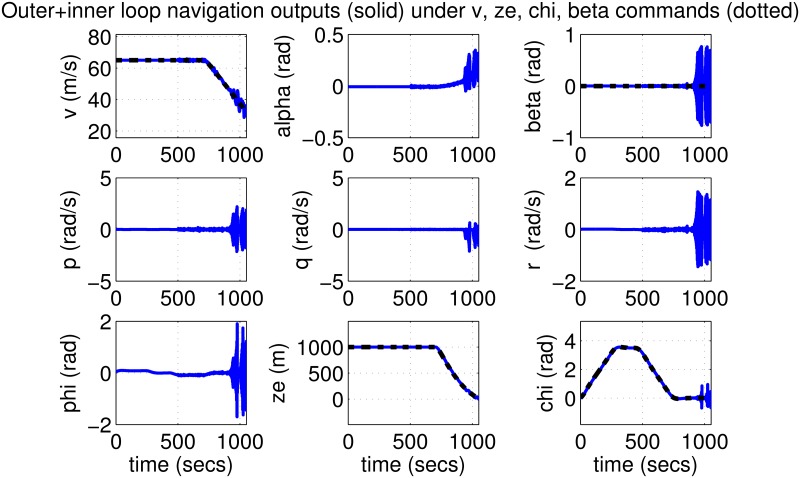
States of a 30% perturbed aircraft with inner/outer loop-shaping control during final test scenario.

**Fig 24:**
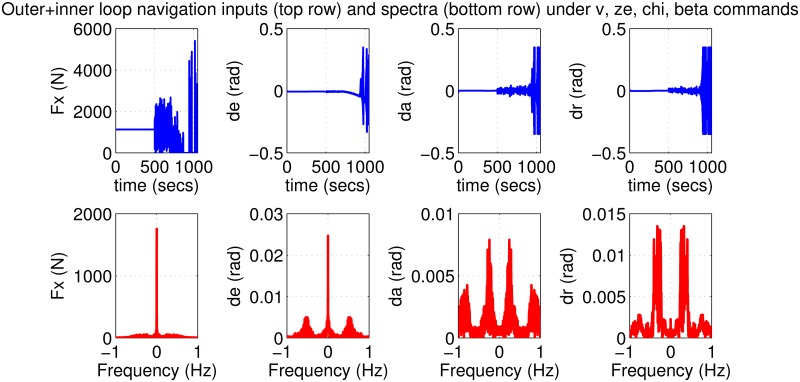
Inputs to a 30% perturbed aircraft with inner/outer loop-shaping control during final test scenario.

**Fig 25:**
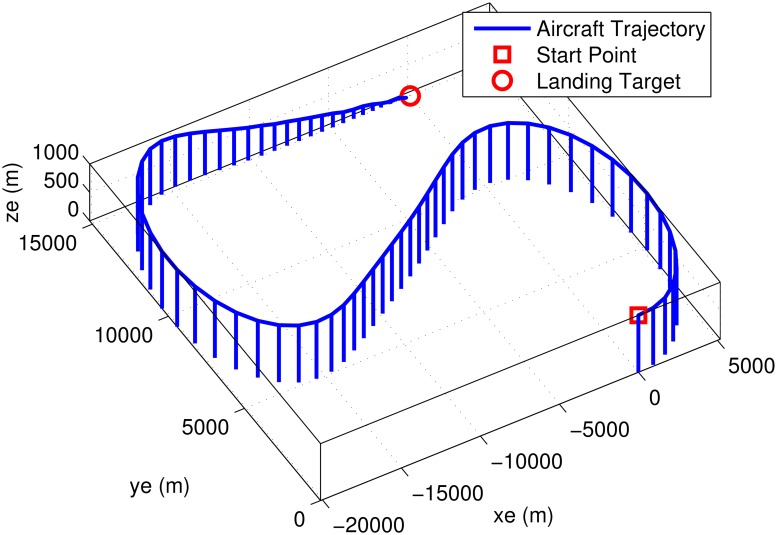
Trajectory of a 30% perturbed aircraft with inner/outer loop-shaping control during final test scenario.

The initial test is performed using pulse-type references both on the nominal model and on perturbed models with up to 30% uncertainty. Plots in Figs 19–22 indicate good performance for this situation. Finally, SIL simulations were performed with the flight controller implemented in MATLAB/Simulink and the blade element simulations carried out by the flight simulator X-Plane. The former sends thrust, elevator, aileron, rudder commands to the latter and receives flight simulation results in real-time every 25 ms through user datagram protocol (UDP) packets (Fig 5). Numerous scenarios with up to 30% perturbation were studied in this configuration with success and one is presented here as an example. In this case a perturbed Cessna 172 cruising at 65 m/s and 1000 m receives appropriate commands for maneuvers required to navigate to a target airfield, line-up with the runway, descend, flare and touchdown. *r*_*β*_ is always zero for turn coordination and flight comfort. For the first 500 seconds the weather remains calm but after *t* = 500 s, the wind magnitude is steadily increased, reaching 15 m/s during landing phase with the crosswind component reaching 7 m/s. Some oscillation in the states are unavoidable for high winds; nevertheless the flight control system responds well to the commands as seen in Fig 23. The control inputs applied to the aircraft and their frequency spectra is presented in Fig 24, which are within practical limits. The 3D trajectory is also visualized in Fig 25. Overall the flight control system maintains stable flight, responds well to commands received and executes the landing, even under unfavorable atmospheric conditions.

*Remark*. The careful reader might notice that the thrust *F*_*x*_ drops to zero around *t* = 800 s in Fig 24. This corresponds to the throttle being cut, which is a normal event within the landing phase. When the aircraft is higher than desired during landing, reducing/cutting the throttle is typical procedure; conversely the throttle is boosted if the is the aircraft is too low. Also recall that strong winds are present during the landing phase. Thus the aircraft can be suddenly pushed upward or downward by wind gusts, calling for a rapid correction from the autopilot. A similar reasoning can be made for the other control surfaces. It should be noted however that the long duration for the simulation (about 1000 s) compresses the plotted data and gives the false illusion that the fluctuations are quite wild. Observing the frequency responses in the bottom row of the figure reveals that the control inputs do not contain significant power at frequencies higher than about 0.5 Hz. This means that the autopilot does not cause any sharp thrust changes or wild oscillations in the control surfaces.

## Conclusions

Two multi-input multi-output (MIMO) control design approaches were investigated to handle parametric uncertainties in autopilot design for aircrafts. In real-life it is impossible to perfectly determine geometry, mass and stability derivative parameters so some level of uncertainty is inevitable. The study was carried out on the aircraft dynamics as a whole and not on individual modes, which is essential to capture all the coupling effects. It was revealed that an attitude controller working perfectly on individual nominal models can lose stability with a perturbation as small as 2%. Robust MIMO design using loop-shaping and *μ*-synthesis were presented as remedies, which were seen to withstand high parametric variations of 30%, while retaining good performance. As a final test, the attitude controller was augmented with an outer loop controller designed also using loop-shaping, forming a complete flight control system. It was confirmed through software-in-the-loop (SIL) verifications using blade element theory (BET) that the autopilot is capable of performing navigation and landing under high parametric variations and strong winds.

Currently work is in progress for establishing hardware-in-the-loop (HIL) and flight test platforms to validate the outlined design strategy [58–67]. Future research directions also include studying alternative methods for the controllers and investigating the possibility of reducing controller size.

## Supporting Information

S1 File Dataset. This file contains MATLAB data and functions for the results in the article.
(RAR)

## References

[1] Etkin B, Reid LD. Dynamics of flight: stability and control, 3rd edition New York: Wiley; 1996.

[2] Nelson RC. Flight stability and automatic control. McGraw Hill; 1998.

[3] Stevens BL, Lewis FL. Aircraft Control and Simulation, 2nd edition Wiley-Interscience; 2003.

[4] Blakelock JH. Automatic control of aircraft and missiles, John Wiley and Sons; 1991.

[5] Chao H, Cao Y, Chen Y. Autopilots for small unmanned aerial vehicles: a survey. International Journal of Control, Automation and Systems. 2010;8(1):36–44. 10.1007/s12555-010-0105-z

[6] Martin J, Edwards HH, Burgess MA, Percival HF, Fagan DE, Gardner BE, et al Estimating distribution of hidden objects with drones: From tennis balls to manatees. PLoS One. 2012;7(6):e38882 10.1371/journal.pone.0038882 22761712 PMC3382610

[7] Rodríguez A, Negro JJ, Mulero M, Rodríguez C, Hernández-Pliego J, Bustamante J. The eye in the sky: combined use of unmanned aerial systems and GPS data loggers for ecological research and conservation of small birds. PLoS One. 2012;7(12):e50336 10.1371/journal.pone.0050336 23239979 PMC3519840

[8] Chabot D, Craik SR, Bird DM. Population census of a large common tern colony with a small unmanned aircraft. PloS one. 2015;10(4):e0122588 10.1371/journal.pone.0122588 25874997 PMC4398491

[9] Reiner J, Balas GJ, Garrard WL. Flight control design using robust dynamic inversion and time-scale separation. Automatica. 1996;32(11):1493–1504. 10.1016/S0005-1098(96)00101-X

[10] Murillo MH, Limache AC, Fredini PSR, Giovanini LL. Generalized nonlinear optimal predictive control using iterative state-space trajectories: Applications to autonomous flight of UAVs. International Journal of Control, Automation and Systems. 2015;13(2):361–370. 10.1007/s12555-013-0416-y

[11] Calise AJ, Lee S, Sharma M. Development of a reconfigurable flight control law for tailless aircraft. Journal of Guidance, Control, and Dynamics. 2001;24(5):896–902. 10.2514/2.4825

[12] Wang Q, Stengel RF. Robust nonlinear control of a hypersonic aircraft. Journal of Guidance, Control, and Dynamics. 2000;23(4):577–585. 10.2514/2.4580

[13] Lawrence DA, Frew EW, Pisano WJ. Lyapunov vector fields for autonomous unmanned aircraft flight control. Journal of Guidance, Control, and Dynamics. 2008;31(5):1220–1229. 10.2514/1.34896

[14] Du R, Meng K, Zhou D, Liu J. Design of three-dimensional nonlinear guidance law with bounded acceleration command. Aerospace Science and Technology. 2015;46:168–175. 10.1016/j.ast.2015.07.010

[15] Liu X, Liu Z, Shan J, H S. Anti-disturbance autopilot design for missile system via finite time integral sliding mode control method and nonlinear disturbance observer technique. Transactions of the Institute of Measurement and Control. 2015;1(1):0142331215603793.

[16] Athans M, Castanon D, Dunn KP, Greene CS, Lee WH, Sandell NR, et al The stochastic control of the F-8C aircraft using a multiple model adaptive control (MMAC) method—Part I: Equilibrium flight., IEEE Transactions on Automatic Control. 1977;22(5):768–780. 10.1109/TAC.1977.1101599

[17] Karagiannis D, Astolfi A. Non-linear and adaptive flight control of autonomous aircraft using invariant manifolds. Proceedings of the Institution of Mechanical Engineers, Part G: Journal of Aerospace Engineering. 2010;224(4):403–415.

[18] Gao MZ, Cai GP, Nan Y. Robust adaptive fault-tolerant H-infinity control of reentry vehicle considering actuator and sensor faults based on trajectory optimization. International Journal of Control, Automation and Systems. 2016;14(1):198–210. 10.1007/s12555-014-0457-x

[19] Goodarzi FA, Lee D, Lee T. Geometric control of a quadrotor UAV transporting a payload connected via flexible cable. International Journal of Control, Automation and Systems. 2015;13(6):1486–1498. 10.1007/s12555-014-0304-0

[20] Kang S, Kim HJ, Lee JI, Jun BE, Tahk MJ. Roll-pitch-yaw integrated robust autopilot design for a high angle-of-attack missile. Journal of guidance, control, and dynamics. 2009;32(5):1622–1628. 10.2514/1.39812

[21] Choi B, Kang S, Kim HJ, Jun BE, Lee JI, Tahk MJ, et al Roll-pitch-yaw integrated *μ*-synthesis for high angle-of-attack missiles. Aerospace Science and Technology. 2012;23(1):270–279. 10.1016/j.ast.2011.08.002

[22] Bruyere L, White B, Tsourdos A. Dynamic inversion for missile lateral velocity control via polynomial eigenstructure assignment. In: Proceedings of the AIAA Guidance, Navigation and Control Conference and Exhibit; 2003.

[23] Tsay TS. Decoupling the flight control system of a supersonic vehicle. Aerospace Science and Technology. 2007;11(7):553–562. 10.1016/j.ast.2007.05.005

[24] Basri MAM, Husain AR, Danapalasingam KA. Intelligent adaptive backstepping control for MIMO uncertain non-linear quadrotor helicopter systems. Transactions of the Institute of Measurement and Control. 2014;1(1):0142331214538900.

[25] Yang CD, Liu WH. Nonlinear H-infinity decoupling hover control of helicopter with parameter uncertainties. American Control Conference; 2003.

[26] Shouzhao S, Chenwu S, Haibin D, Xiaoliang J, Yansong Z. Longitudinal and lateral adaptive flight control design for an unmanned helicopter with coaxial rotor and ducted fan. 33rd Chinese Control Conference (CCC); 2014.

[27] Ozbek NS, Onkol M, Efe MO. Feedback control strategies for quadrotor-type aerial robots: a survey. Transactions of the Institute of Measurement and Control. 2015;1(1):0142331215608427.

[28] Liu H, Li D, Kim J, Zhong Y. Real-Time Implementation of Decoupled Controllers for Multirotor Aircrafts. Journal of Intelligent & Robotic Systems. 2014;73:197–207. 10.1007/s10846-013-9916-5

[29] Ahmad SM, Chipperfield AJ, Tokhi MO. Parametric modelling and dynamic characterization of a two-degree-of-freedom twin-rotor multi-input multi-output system. Proceedings of the Institution of Mechanical Engineers, Part G: Journal of Aerospace Engineering. 2001;215(2):63–78.

[30] Efe MO. Integral sliding mode control of a quadrotor with fractional order reaching dynamics. Transactions of the Institute of Measurement and Control. 2010;33(8):985–1003. 10.1177/0142331210377227

[31] de Voogt AJ, van Doorn RRA. Accidents associated with aerobatic maneuvers in US aviation. Aviation, space, and environmental medicine. 2009;80(8):732–733. 10.3357/ASEM.2521.2009 19653578

[32] Wong DK, Pitfield DE, Caves RE, Appleyard AJ. Quantifying and characterising aviation accident risk factors. Journal of Air Transport Management. 2006;12(6):352–357. 10.1016/j.jairtraman.2006.09.002

[33] Lykins R, Keshmiri S, Riley R, Garcia G. Modal Analysis of 1/3-Scale Yak-54 Aircraft Through Simulation and Flight Testing. In: AIAA Atmospheric Flight Mechanics Conference; 2011. p. 6443.

[34] Royer D, Keshmiri S, Sweeten B, Jones V. Modeling and sensitivity analysis of the meridian unmanned aircraft AIAA Infotech@ Aerospace. 2010;.

[35] Schueler CJ, Ward LK, Hodapp A Jr. Techniques for measurement of dynamic stability derivatives in ground test facilities DTIC Document; 1967.

[36] Nae C. VLM Tool for IDS Integration. INCAS Bulletin. 2010;2(1):37–48. 10.13111/2066-8201.2010.2.1.5

[37] Gill SJ, Lowenberg MH, Neild SA, Crespo LG, Krauskopf B, Puyou G. Nonlinear Dynamics of Aircraft Controller Characteristics Outside the Standard Flight Envelope. Journal of Guidence, Control, and Dynamics. 2015;38(12):2301–2308. 10.2514/1.G000966

[38] Ravanbod L, Noll D. Gain-scheduled two-loop autopilot for an aircraft. Journal of Dynamic Systems, Measurement, and Control. 2014;136(4):041021 10.1115/1.4026832

[39] Mendez-Vergara F, Cervantes I, Mendoza-Torres A. Stability of gain scheduling control for aircraft with highly nonlinear behavior. Mathematical Problems in Engineering. 2014;2014.

[40] Stengel RF. Some effects of parameter variations on the lateral-directional stability of aircraft. Journal of Guidance, Control, and Dynamics. 1980;3(2):124–131. 10.2514/3.55959

[41] Ingram G. Wind Turbine Blade Analysis using the Blade Element Momentum Method, Durham, UK: School of Engineering. Durham University; 2005.

[42] Burton T, Sharpe D, Jenkins N, Bossanyi E. Wind energy handbook. West Sussex: John Wiley & Sons; 2001.

[43] Rauw MO. FDC 1. 2—A Simulink Toolbox for Flight Dynamics and Control Analysis, Haarlem, The Netherlands; 2001.

[44] Leisher L, Walter H. Stability derivatives of cessna aircraft. Cessna Aircraft Company. 1957;7.

[45] Neuhart RA, Gingras DR, Hultberg RS, Oltman RS, Graybeal NW. Flight Data Collection for General Aviation Aircraft Simulation Validation. In: AIAA Atmospheric Flight Mechanics Conference; 2009. p. 5731.

[46] Joint Aviation Authorities. Joint Aviation Requirements: JAR-STD 1A Aeroplane Flight Simulators Amendment 3; 2003. JAR STD 1A.

[47] Federal Aviation Administration. Flight Simulation Training Device Initial and Continuing Qualification and Use; Final Rule; 2008. FAA 14 CFR Part 60.

[48] Fletcher R. Practical Methods of Optimization, New York: John Wiley and Sons; 1987.

[49] Schittkowski K. NLPQL: A FORTRAN subroutine solving constrained nonlinear programming problems. Annals of operations research. 1986;5(2):485–500. 10.1007/BF02739235

[50] Le V, Safonov MG. Rational matrix GCDs and the design of squaring-down compensators-a state-space theory. Automatic Control, IEEE Transactions on. 1992;37(3):384–392. 10.1109/9.119644

[51] Chen M, Desoer C. Necessary and sufficient condition for robust stability of linear distributed feedback systems. International Journal of Control. 1982;35(2):255–267. 10.1080/00207178208922617

[52] Glover K, McFarlane D. Robust stabilization of normalized coprime factor plant descriptions with H-bounded uncertainty. Automatic Control, IEEE Transactions on. 1989;34(8):821–830. 10.1109/9.29424

[53] Doyle J. Structured uncertainty in control system design. In: 1985 24th IEEE Conference on Decision and Control. 24; 1985. p. 260–265.

[54] Zhou K, Doyle JC, Glover K, et al Robust and optimal control. vol. 40 Prentice Hall New Jersey; 1996.

[55] Doyle JC, Glover K, Khargonekar PP, Francis BA. State-space solutions to standard H 2 and H control problems. IEEE Transactions on Automatic control. 1989;34(8):831–847. 10.1109/9.29425

[56] Ho WK, Hang CC, Zhou JH. Performance and gain and phase margins of well-known PI tuning formulas. Control Systems Technology. 1995;3(2):245–248. 10.1109/87.388135

[57] Chien IL. Consider IMC tuning to improve controller performance. Chem Eng Prog. 1990;86:33–41.

[58] Atlas E, Erdoğan MI, Ertin OB, Güçlü A, Saygi YE, Kaynak Ü, et al Hardware-in-the-Loop Test Platform Design for UAV Applications In: Applied Mechanics and Materials. vol. 789 Trans Tech Publ; 2015 p. 681–687.

[59] Kasnakoglu C, Kaynak U. Automatic Recovery and Autonomous Navigation of Disabled Aircraft After Control Surface Actuator Jam. AIAA Guidance, Navigation and Control Conference, Toronto, Canada; 2010.

[60] Kaynak U, Akbaba R, Kibar A, Kasnakoglu C, Sezer-Uzol N, Gulec E, et al. Design and Manufacture of a Fuel Cell Powered Unmanned Air Vehicle. In: International Unmanned Vehicles Workshop UVW2010. Istanbul, Turkey; 2010.

[61] Korkmaz H, Ertin OB, Kasnakoglu C, Kaynak U. Design of a Flight Stabilizer System for a Small Fixed Wing Unmanned Aerial Vehicle Using System Identification. IFAC Workshop on Advances in Control and Automation Theory for Transportation Applications (ACATTA 2013), Istanbul, Turkey; 2013.

[62] Ertin OB, Korkmaz H, Kaynak U, Kasnakoglu C. Hardware-in-the-Loop Test Platform for a Small Fixed Wing Unmanned Aerial Vehicle Embedded Controller. The 32nd IASTED International Conference on Modelling, Identification and Control (MIC 2013), Innsbruck, Austria; 2013.

[63] Akyürek Ş, Özden GS, Kürkçü B, Kaynak Ü, Kasnakoğlu C. Design of a flight stabilizer for fixed-wing aircrafts using H loop shaping method. In: 9th International Conference on Electrical and Electronics Engineering (ELECO). IEEE; 2015. p. 790–795.

[64] Akyürek Ş, Kürkçü B, Kaynak Ü, Kasnakoğlu C. Control Loss Recovery Autopilot Design for Fixed-Wing Aircraft. IFAC-PapersOnLine. 2016;49(9):117–123. 10.1016/j.ifacol.2016.07.509

[65] Akyurek S, Kaynak U, Kasnakoglu C. Altitude Control for Small Fixed-Wing Aircraft Using H Loop-Shaping Method. IFAC-PapersOnLine. 2016;49(9):111–116. 10.1016/j.ifacol.2016.07.507

[66] Kasnakoğlu C. Scheduled smooth MIMO robust control of aircraft verified through blade element SIL testing. Transactakyurek2016designions of the Institute of Measurement and Control. 2016; p. 0142331216661760.

[67] Akyürek S, Özden GS, Atlas E, Kasnakoglu C, Kaynak Ü. Design of a Flight Stabilizer System and Automatic Control Using HIL Test Platform. International Journal of Mechanical Engineering and Robotics Research. 2016;5(1):77.

